# Method for quick DNA barcode reference library construction

**DOI:** 10.1002/ece3.7788

**Published:** 2021-08-04

**Authors:** Yanlei Liu, Chao Xu, Yuzhe Sun, Xun Chen, Wenpan Dong, Xueying Yang, Shiliang Zhou

**Affiliations:** ^1^ State Key Laboratory of Systematic and Evolutionary Botany Institute of Botany Chinese Academy of Sciences Beijing China; ^2^ College of Life Sciences University of Chinese Academy of Sciences Beijing China; ^3^ College of Landscape Architecture Northeast Forestry University Harbin China; ^4^ Laboratory of Systematic Evolution and Biogeography of Woody Plants School of Ecology and Nature Conservation Beijing Forestry University Beijing China; ^5^ National Engineering Laboratory for Forensic Science Key Laboratory of Forensic Genetics Institute of Forensic Science Ministry of Public Security Beijing China

**Keywords:** Cotu, data processing method, DNA barcode, next‐generation sequencing

## Abstract

DNA barcoding has become one of the most important techniques in plant species identification. Successful application of this technology is dependent on the availability of reference database of high species coverage. Unfortunately, there are experimental and data processing challenges to construct such a library within a short time. Here, we present our solutions to these challenges. We sequenced six conventional DNA barcode fragments (ITS1, ITS2, *matK*1, *matK*2, *rbcL*1, and *rbcL*2) of 380 flowering plants on next‐generation sequencing (NGS) platforms (Illumina Hiseq 2500 and Ion Torrent S5) and the Sanger sequencing platform. After comparing the sequencing depths, read lengths, base qualities, and base accuracies, we conclude that Illumina Hiseq2500 PE250 run is suitable for conventional DNA barcoding. We developed a new “Cotu” method to create consensus sequences from NGS reads for longer output sequences and more reliable bases than the other three methods. Step‐by‐step instructions to our method are provided. By using high‐throughput machines (PCR and NGS), labeling PCR, and the Cotu method, it is possible to significantly reduce the cost and labor investments for DNA barcoding. A regional or even global DNA barcoding reference library with high species coverage is likely to be constructed in a few years.

## Graphical abstract

In this study, two next‐generation sequencing platform and four data processing methods were compared for further DNA barcoding reference library construction. After comparing the sequencing depths, read lengths, base qualities, and base accuracies, we concluded that Illumina Hiseq2500 PE250 run is suitable for conventional DNA barcoding, and after comparing our new “Cotu” to other three NGS data processing methods, we recommend Cotu method for its most sequence recovery, the longest sequence length, and the highest sequence accuracy. By using herbarium materials, high‐throughput machines (PCR and NGS), and the Cotu method, it is possible to construct a regional or even global DNA barcoding reference library with high species coverage with limited cost and labor investments in a few years.

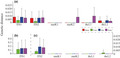

## 1 INTRODUCTION

Since the term “DNA barcode” was proposed (Hebert et al., 2003), DNA barcoding has soon become a routine technology in molecular identification of organisms and served as a new tool for biologists to understand biota (Kress, 2017). This technology has extensive applications for the identification of microorganisms (Barberán et al., 2015), dietary composition of animals (Kartzinel et al., 2015), components in processed foods or drugs (Nithaniyal et al., 2017), cryptic species discovery (Tyagi et al., 2019), invasive species monitor (Xu et al., 2018), rare and endangered species conservation (Giovino et al., 2016; Hosein et al., 2017), etc.

Reliable molecular identification depends on the resolution of molecular markers (or DNA barcodes) and the species coverage of the reference library. Considerable efforts have been made to find the ideal DNA barcodes for plants (CBOL Plant Working Group, 2009; Dong et al., 2014, 2015; Kress & Erickson, 2007; Li et al., 2011) as well as to develop new technical improvements (Giovino et al., 2020a; Hollingsworth et al., 2011; Xu et al., 2015; Yu et al., 2011). Unlike animals which COI (mitochondrial cytochrome oxidase I) is a nearly sole DNA barcode, plant DNA barcoding is much more complicated and no ideal DNA barcodes for plants have yet been discovered, or perhaps they do not exist at all (Giovino et al., 2020b). A well‐curated reference sequence library with high species coverage remains to be constructed for extensive applications of this technology. The good news is that some ambitious projects have been launched in the past few years [e.g., BARCODE 500K (https://ibol.org), BIOSCAN (Hobern & Hebert, 2019), and ISHAM‐ITS (Irinyi et al., 2016)]. Even so, sequence data deposited in public databases are still rather small. Taking 258,650 flowering plants as an example (Thorne, 2002), 51,132 (19.8%) species have *matK* sequences and 46,130 (17.8%) species have *rbcL* sequences deposited in GenBank (accessed on 1 May 2020).

In order to construct such a reference library in a relatively short time, we have to use cost‐efficient next‐generation sequencing (NGS) platforms and acquire the ability of manipulating the NGS data. Although NGS platforms have been applied for this purpose for a decade (Boyer et al., 2016; Piry et al., 2012; Richardson et al., 2017; Shi et al., 2018; Shokralla et al., 2012; Toju, 2015), no consensus has been reached concerning the platforms themselves and the data processing methods owing to rapid replacements or upgrades of sequencing machines. Roche 454, which was one of the most suitable choices for DNA barcoding (Guo et al., 2019; Hajibabaei et al., 2011; Shokralla et al., 2014), is no longer available. The third‐generation sequencing (TGS) or single molecule sequencing (SMS) platforms, such as PacBio and Nanopore, are now available for DNA super barcodes (such as Zhang et al., 2020). Illumina systems (Hiseq and Miseq) and Ion Torrent systems are currently the mainstream NGS platforms for conventional DNA barcode sequencing. The paired‐end 250 (PE250) Illumina Hiseq recovers half the sequence lengths (ca 400 bp after removal of prefixes such as primers) of conventional DNA barcodes of 600–800 bp, whereas the Ion Torrent S5 series has a capacity of generating ca. 600‐bp sequences. Both platforms have been used on DNA metabarcoding of environmental samples (Deagle et al., 2013; Evans et al., 2016; Fantini et al., 2015; Schmidt et al., 2013). The operational taxonomic units (OTUs) from the environmental samples were much more than actual situations, and it is one major concern whether these two platforms are suitable for conventional DNA barcodes or not (Lahens et al., 2017; Marine et al., 2020; Quail et al., 2012; Speranskaya et al., 2018).

Although researchers have made some efforts on applying NGS platforms to conventional DNA barcoding (Akankunda et al., 2020; Creedy et al., 2020; de Kedrel et al., 2020; Srivathsan et al., 2018), the ease of data analyses is still the other major concern in DNA barcoding using NGS. Several software packages have been developed for DNA metabarcoding, such as Vsearch, Usearch, Qiime2, OBITools, PipeCraft, and Mothur (Anslan et al., 2017; Boyer et al., 2016; Callahan et al., 2016; Edgar, 2013, 2016; Rognes et al., 2016; Schloss et al., 2009). Although these software packages can also be adopted to conventional DNA barcodes, there are some gaps to be bridged in the NGS data analysis pipelines. For example, a DNA metabarcoding sample contains many species, whereas in conventional DNA barcoding, a sample usually contains only one species. For cost efficiency consideration, many gene fragments of multiple samples are DNA‐labeled, mixed, and sequenced in a single NGS run. Demultiplexing is necessary, and usually, only one or a few sequences need to be generated for each gene fragment in each sample.

One of the outstanding features of NGS is its ability to generate large data and different software packages with different functions have to be used. The NGS data processing major include (a) quality control to find and remove reads of low quality; (b) assembling read1 and read2 when paired‐end sequencing method is used; (c) demultiplexing data to assign data to genes of samples according to primer and label sequences; and (d) creating correct consensus sequences when using NGS for conventional DNA barcode creation. Unfortunately, most scientists who devote themselves to DNA barcoding do not have the skills to handle such kind of data.

There are two prevailing strategies for processing amplicon sequencing data from NGS platforms. The first one applies an arbitrary cutoff (say, a minimum similarity of 0.97) for lumping reads into OTUs. Software packages, such as Usearch, Vsearch, Mothur, and PipeCraft, use this strategy. The second strategy resolves amplicon sequence variants (ASVs) to proofread the final sequences based on the sequencing depth of exact sequence variants (ESVs, usually one nucleotide difference; Knight et al., 2018). Software packages DADA2 and Unoise3 belong to this strategy (Callahan et al., 2017; Edgar, 2018). This strategy avoids imposing the arbitrary similarity thresholds but introduces random sequencing errors because not all single base differences are real. For the final sequence generation, Usearch, Mothur, Swarm, DADA2, etc., pick up a representative sequence while Vsearch, Geneious, Mothur, etc., create a consensus sequence. The representative sequence strategy has a risk of introducing sequencing errors and length variations to the final sequences. The consensus sequence strategy retains indel errors in homopolymeric regions (Srivathsan et al., 2018) and sequence length variations at both sequence ends. Current software is still imperfect for creating DNA barcodes, and they were not designed specially for conventional DNA barcode data analysis. In order to improve the accuracy and length of output consensus sequences, we developed the new Cotu method under the majority rule (the letter “C” stands for consensus, Cotu means consensus‐based OTU creation method, and the majority rule means only the majority base will be treated as the right base in each certain position in an alignment file).

Although a few studies have made performance comparisons between Illumina and Ion Torrent sequencing platforms (Lahens et al., 2017; Salipante et al., 2014; Speranskaya et al., 2018), it is still difficult for researchers to determine with certainty which one is more suitable for DNA barcoding. Similarly, due to imperfections of current data processing software, it is still unknown to researchers which software is most reliable in creating final sequences. In this study, we tested the suitability of two popular NGS platforms, Illumina Hiseq2500 and Ion Torrent S5, based on (a) the base quality; (b) read length variation; (c) sequencing depth bias among samples; and (d) genetic distance. For data processing methods, we compared the reliability of final sequences created by three most widely used data analysis methods (Otu, Zotu, and DADA2) to the standard sequences obtained from Sanger sequencing platform and provided our solution Cotu method in creating final sequences. The parameters we used are (a) sequence recovery; (b) sequence length; (c) genetic distance; and (d) sequence reliability. Among the four methods for clustering reads, Otu and Cotu use cutoff parameter, and Zotu and DADA2 use ESV strategy. Otu, Zotu, and DADA2 methods generate sequences using the representative sequence principle while Cotu adopts a majority consensus strategy. We aim to provide a better methodological solution to the collection of reliable sequences using NGS for the construction of a DNA barcode reference library in a short time with investments as small as possible.

## 2 MATERIALS AND METHODS

**FIGURE 1:**
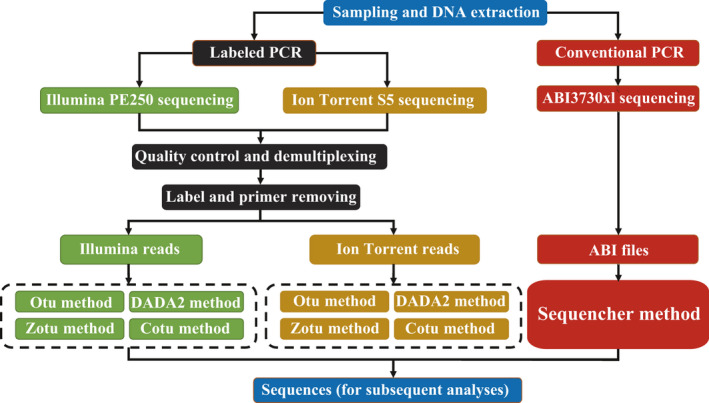
Experimental workflow. Gene fragments are sequenced on ABI 3730xl, Illumina Hiseq2500, and Ion Torrent S5 platforms. Data generated by the latter two platforms are processed with Cotu, Dotu, Otu, and Zotu methods. The sequences from ABI 3730xl serve as references and those from the two NGS platforms are queries in analyses

Our experimental workflow is depicted in Figure 1. Gene fragments were sequenced by the Sanger sequencing method on ABI 3730xl, and NGS method on Illumina Hiseq2500 PE250 and Ion Torrent S5xl platforms. The clean reads from the Illumina and Ion Torrent platforms were analyzed using Otu, Zotu, DADA2, and Cotu methods. Sequences from ABI 3730xl were used as “gold standards” to test the results of the four methods using the reads from the two NGS platforms (details shown below).

### 2.1 Plant material sampling and DNA extraction

All materials were collected from Beijing Botanical Garden of the Institute of Botany, Chinese Academy of Sciences. Fresh leaf materials of 380 samples (Table S1) belonging to 253 species, 139 genera, and 60 families were collected and immediately oven‐dried at 65℃ for 2–3 hr. Most species were collected during their flowering period. One to five individuals were sampled for each species. All voucher specimens were deposited in the herbarium of Institute of Botany, the Chinese Academy of Sciences (PE). Total DNA was extracted using the mCTAB method (Li et al., 2013), and the concentration was adjusted to 10 ng/μl for subsequent PCR experiments according to the measurements of the Nanodrop 2000c spectrophotometer (Thermo Fisher Scientific Inc.).

### 2.2 PCR for Sanger sequencing

The nuclear internal transcribed spacer (ITS), chloroplast maturase K (*matK*), and ribulose‐1,5‐bisphosphate carboxylase/oxygenase (*rbcL*) were amplified using universal primers ITS‐P5 + ITS‐U4, matK‐472F + matK‐1248R, and rbcLbF + rbcLbR (Table 1) for Sanger sequencing (Dong et al., 2015). *psbA‐trnH* was not used due to a long homopolymeric region frequently existing in many samples. Fragments were sequenced on ABI 3730xl DNA Analyzer (Applied Biosystems, USA) at the Majorbio Company in Beijing, China.

**TABLE 1:** The first‐round PCR primers for amplifying DNA fragments

Barcode	Primer name	Primer sequence (5'−3')	Tm(℃)	GC%	Expected length
ITS1	ITS‐P5	CCTTATCAYTTAGAGGAAGGAG	68.16	47.22	370−380 bp
	ITS‐U2	GCGTTCAAAGAYTCGATGRTTC	68.73	47.22	
ITS2	ITS‐P3	YGACTCTCGGCAACGGATA	69.70	54.55	440−450 bp
	ITS‐U4	RGTTTCTTTTCCTCCGCTTA	67.15	47.06	
*matK*1	*matK*−472F	CCCRTYCATCTGGAAATCTTGGTTC	70.54	48.72	380−390 bp
	*matK*−821R	TTTCCTTGATATCTAACATAATG	64.20	37.84	
*matK*2	*matK*−821F	CATTATGTTAGATATCAAGGAAA	64.20	37.84	420−430 bp
	*matK*−1248R	GCTRTRATAATGAGAAAGATTTCTGC	67.32	44.00	
*rbcL*1	*rbcL*bF	AGACCTWTTTGAAGAAGGTTCWGT	67.50	44.74	420−430 bp
	*rbcL*717R	CATGTACCTGCAGTAGCATTCAAGT	69.49	48.72	
*rbcL*2	*rbcL*717F	ACTTGAATGCTACTGCAGGTACATG	69.49	48.72	430−440 bp
	*rbcL*bR	TCGGTYAGAGCRGGCATRTGCCA	72.51	56.76	

### 2.3 PCR for next‐generation sequencing (NGS)

**FIGURE 2:**
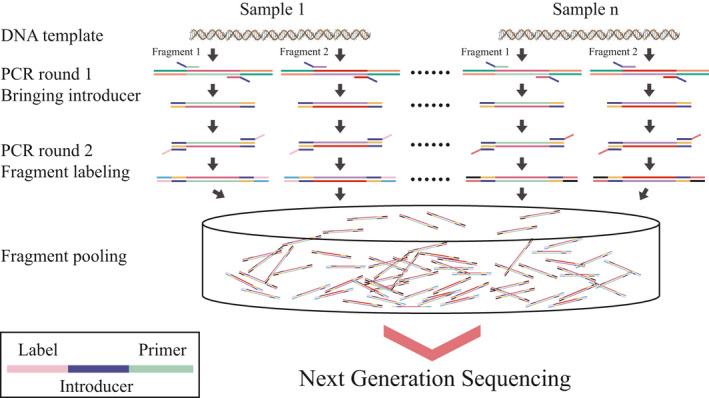
Labeling gene fragments using sample‐specific oligoes by two rounds of PCR for multiplexed sequencing on the Illumina Hiseq2500 and Ion Torrent S5 platforms. An “introducer” is attached to the ends of fragments during the first round of PCR. The introducer serves as the priming site during the second round of PCR, and sample‐unique oligoes were added to the ends of the fragments

In order to meet the read length limitations of Illumina and Ion torrent S5 sequencing platforms, we amplified two fragments about 400 bp for each conventional barcode (middle primers were used and listed in Table 1 and the primer annealing positions are displayed in Figure S1). When designing the middle primers, overlap region was considered for the whole ITS assembling. The length of DNA barcode *matK* and *rbcL* is about 800 bp each, and they are very variable among angiosperm plants. It is hard to find two possible positions to design primer pairs forming overlaps in the middle of the DNA barcodes. In order to improve the primer universality, we only find one position for *matK* and *rbcL* each to design the overlapped primers. Therefore, two parts of each DNA barcode can also be assembled through the overlap positions of the middle primers. For multiplexing on NGS platforms, gene fragments from the same sample were labeled with a unique DNA oligo by two rounds of PCR (Figure 2). In the first round of PCR, primers attached by an oligo (called introducer, 5’‐GTAGACTGCGTACC‐3’) at the 5’ end were used to amplify gene fragments. The PCR procedures were the same as Dong et al. (2015), except that the primer concentration was only 10% of that in conventional PCR and that the number of PCR cycles was increased to 40 for using up all primer molecules. In the second round of PCR, products from the first‐round PCR served as templates, and the introducer attached by a sample‐specific oligo of ten bases (called DNA label) at the 5’ end was used as a primer for each sample. In the present study, 380 such primers were synthesized and used to label 380 samples (Table S1). Different Gene fragments from the same sample were amplified individually with the same labeling primer. The PCR program was the same as Dong et al. (2015).

The PCR products of the same gene were mixed, gel‐purified, and quantified on a Nanodrop 2000c spectrophotometer. The PCR products of different genes were combined at nearly equal molar ratios according to their concentrations.

### 2.4 Library construction for next‐generation sequencing

The final PCR mixture for NGS was divided into two equal parts. One part was used for Illumina library construction using NEBNext® Ultra™ DNA Library Prep Kit for Illumina® (New England BioLabs) and sequenced at BerryGenomics, Beijing, China, on Illumina Hiseq2500 (pair end, PE250). The other part was used for Ion Torrent S5 platform library construction using NEBNext® Fast DNA Library Prep Set for Ion Torrent (New England BioLabs) and sequenced at Maize Research Center, Beijing Academy of Agriculture and Forestry Sciences for Ion Torrent S5 Chip400 sequencing.

### 2.5 ABI 3730xl sequencing data processing

Sequences were assembled by combining forward and reverse strands of the same fragments according to the overlap region, and they were edited using Sequencher v5.4.5 (Gene Codes Corporation) based on files from ABI 3730xl Analyzer. Base‐calling mistakes if any were corrected according to the chromatograms. If the major base chromatogram peak is obviously bigger than other little chromatograms, we consider this base is correct. If there are two major chromatograms, we consider this base as a degenerate base. If there are no major chromatogram and the chromatogram peak is low, we consider this base is not credible.

### 2.6 Illumina Hiseq2500 and Ion Torrent S5 sequencing data processing

#### 2.6.1 Quality control

Illumina Hiseq2500 PE250 data were quality‐controlled using the NGS QC toolkit v2.3.3 with the default parameters (Patel & Jain, 2012). After quality control, the lengths of the reads longer than 200 bp were categorized by genes and by platforms using the default parameters in FASTX‐Toolkit v0.0.13 (http://hannonlab.cshl.edu/fastx_toolkit/) and statistics was done with Excel 2016.

#### 2.6.2 Demultiplexing and data cleaning

The quality‐controlled reads were merged using Flash v1.2.11 (Magoc & Salzberg, 2011) with default settings (Ion Torrent S5 data processing did not include this step). The merged reads were demultiplexed using FASTX‐Toolkit v0.0.13 (http://hannonlab.cshl.edu/fastx_toolkit/) according to the sample labels and primers. In order to find out which platform had less sequencing bias among samples, the number of reads was transformed into percentages, and the significance of sequencing depth bias variations among samples was tested using double factor variance analysis (two‐way ANOVA) in SPSS v19 between the two platforms gene by gene and as a whole.

Unlabeled sequences and sequences shorter than 200 bp were discarded using NGS QC toolkit v2.3.3. Artificially added regions, such as sample labels, introducers, and primers, were trimmed off using Cutadapt v2.7 (https://cutadapt.readthedocs.io/en/stable/).

#### 2.6.3 Sequencing error estimation

The demultiplexed clean reads of each sample were mapped to the corresponding reference sequences obtained from Sanger sequencing using Geneious Prime 2019.2.3 by the highest sensitivity. The frequencies of the bases along the whole length were calculated using python script *base‐counter.py* (https://github.com/Mycroft‐maker/Base‐counter). Site‐by‐site comparisons were carried out between the final sequences from both platforms and the references from ABI. If there was a mismatch of the highest frequency to the reference, the secondly highest base was considered, and so forth until an exact match was found. The mismatches are an estimate of sequencing errors.

#### 2.6.4 Sequence creation

Four methods (Otu, Zotu, DADA2, and Cotu) were used to create final sequences. For Otu method, we followed the UPARSE protocol (http://www.drive5.com/uparse) (Edgar, 2013; Edgar & Flyvbjerg, 2015). For Zotu method, we followed the Unoise3 protocol (http://www.drive5.com/usearch/manual/unoise_algo) (Edgar, 2013; Edgar & Flyvbjerg, 2015). For DADA2 method, we used the DADA2 (Callahan et al., 2016) plugin in Qiime2 2019.04 (Bolyen et al., 2019) and we call the sequence generated by DADA2 method “Dotu” in this study.

The major features of the Cotu method are elimination of insertions caused by occasional reads under majority rule and sequence length extensions at both ends of alignments. The qualities of the beginning and ending bases are usually low and frequently trimmed off, which causes uneven alignments. If the majority rule was used for this situation, shortened consensus sequences would likely be created. We, therefore, apply a threshold of bases on a site (e.g., 20%) at both ends. In order to reduce gaps in an alignment, base proportion with less than 10% of total bases in a certain position is automatically removed before the application of majority rule. The program calculates the number of bases in three consecutive base positions and sets a position range based on the first appearance of three consecutive base positions with both bases number larger than 50% from each ends automatically. If a site in the beginning and ending regions has the number of bases less than the threshold, the site is considered an incorrect insertion; if a site has bases of more than the threshold number, the site is considered normal and the majority rule is applied. For this method, demultiplexed single copy reads are accurately aligned with Mafft v7.467 (Nakamura et al., 2018) and a consensus sequence is created using Cotu‐Generator.py (https://github.com/YanleiLiu1989/Cotu‐master). For data consisting of multiple copies (such as some nuclear genes and allotetraploids) or multiple species (such as DNA metabarcoding), the clean reads are first sorted into gene copies or species using Vsearch V2.4.3. The sorted reads are then accurately aligned with Mafft v7.467 and a consensus sequence is created using Cotu method. User's manual (Supporting Document S1) and step‐by‐step instructions (Supporting Document S2) are provided for correct use of the method.

### 2.7 Comparative analyses between NGS platforms and among data processing methods

The base quality, read length variation, sequencing depth bias among samples, and base accuracy were used to judge the suitability of Illumina Hiseq2500 and Ion Torrent S5 for conventional DNA barcoding. Base quality was quantified by base scores (values from 1 to 45 given by sequencing machine) using FASTX v0.0.13 and averaged over whole length. The sequencing depths were transformed into relative sequencing depths using percentages of the number of reads each sample to the total reads. Base accuracy was evaluated site by site by comparing to the reference sequences and summarized with Excel.

Sequence recovery, sequence length, and genetic distance were used to test the reliability of Otu, Zotu, DADA2, and Cotu data processing methods. Sequence recovery was the percentage of the number of sequences created by each method compared with the total number of samples with data. The sequence length variations were considered by using the whole‐sequence length.

Genetic distance was parameterized using Kimura two‐parameter genetic distance between sequences created by each method (queries) and the corresponding reference sequence of the same sample using Mega 7.0.18(Kumar et al., 2018). All ambiguous sites were ignored for each sequence pair. Only samples with all four methods results and Sanger reference were adopted for genetic distance comparison.

All significance tests of difference between sequencing platforms and among data processing methods were simplified to be one‐factor analyses of variance (ANOVA) in SPSS v19. The original data were transformed into percentages or genetic distances. The percentages or genetic distances were further transformed into square root values to meet the statistical requirement of normal distribution for ANOVA.

## 3 RESULTS

### 3.1 Reference sequences

Among the 380 samples, ITS, *matK*, and *rbcL* fragments were successfully amplified and sequenced in 304, 367, and 369 samples, respectively, with high base quality. The sequence lengths were from 450 bp to 784 bp for ITS (including 5.8S ribosomal RNA sequences), from 652 bp to 754 bp for *matK*, and 785 bp for *rbcL*.

### 3.2 Differences between Illumina Hiseq and Ion Torrent S5 platforms

#### 3.2.1 Sequencing depth

The average sequencing depth of the samples on the Illumina Hiseq platform was 572× (× represents number of reads) for ITS1, 288× for ITS2, 2,850× for *matK*1, 2,234× for *matK*2, 547× for *rbcL*1, and 321× for *rbcL*2 (Figure S2a). The average sequencing depth per sample was 1,135×. The average sequencing depth of the samples on the Ion Torrent S5 platform was 335× for ITS1, 131× for ITS2, 417× for *matK*1, 1,034× for *matK*2, 523× for *rbcL*1, and 737× for *rbcL*2 (Figure S2b). The average sequencing depth per sample was 530×.

#### 3.2.2 Sequencing depth bias among samples

Illumina Hiseq2500 and Ion Torrent S5 exhibited sequencing depth bias among samples. The variances of sequencing depths among samples on Illumina Hiseq2500 were smaller than on Ion Torrent S5 for all six gene fragments (Figure S3). The difference of sequencing depth bias between the two platforms was tested to be significant (*p* <.001, Table 2).

**TABLE 2:** Statistical *F* test of sequencing depth bias, read length, and base accuracy between sequencing platforms

	Sequencing depth bias	Read length	Base accuracy
Mean	0.0026	383.7094	0.9612
Variance	0.0006	6.86247E−05	0.0013
*F*‐value	58.5202	47.3708	81.2693
*p*	<0.001	<0.001	<0.001

Note

Sequencing depth bias is proportion of reads each sample to the total number of reads from a platform. (Paired end) Read length was number of nucleotides. Base accuracy is proportion of correct bases to the total bases.

#### 3.2.3 Read length

For Illumina PE250 run (maximum fragment length 250 bp), the average lengths of reads were 248 bp and 99.1% of read1 and 98.8% of read2 had minimum lengths of 200 bp (Figure S4a). For Ion Torrent S5 400 chip, the average length of reads was 350 bp and 65.4% of the reads had lengths longer than 320 bp (Figure S4b). The read length difference between the two platforms was tested to be significant (*p* < .001, Table 2).

Considerable read length variations were observed in ITS1, ITS2, *rbcL*1, and *rbcL*2 fragments on both platforms with standard deviation from 101.64 to 147.35 (Figure S5, paired‐end reads for Illumina Hiseq2500). The percentages of mean values to the expected lengths ranged from 62% to 93% for Illumina Hiseq2500 and from 73% to 84% for Ion Torrent S5. The averages of the percentages were nearly the same, 78% for Illumina Hiseq2500 and 77% for Ion Torrent S5.

#### 3.2.4 Base quality

The average base quality scores of read1 and read2 from Illumina platform were 38.76 (*SD* = 0.9132) and 38.05 (*SD* = 1.2667), respectively (Figure S6a,b). The quality of read1 was better than that of read2. The average base quality score of Ion Torrent S5 sequences was 25.64 (*SD* = 5.8194, Figure S6c). Base quality decreased with the progress of sequencing on both platforms.

#### 3.2.5 Base accuracy

Both platforms had over 96% matches for *matK*1, *matK*2, *rbcL*1, and *rbcL*2 fragments and relatively poor matches for ITS1 and ITS2 regions (Figure S7). The base accuracy seemed gene fragment‐dependent. Illumina platform performed better than Ion Torrent for ITS1, ITS2, and *rbcL*1, but worse for *matK*1, *matK*2, and *rbcL*2. Mismatches occurred in the more variable regions of ITS1 and ITS2 and in the beginning parts of *matK*1, *matK*2, and *rbcL*1 (Figure S8). The difference of base accuracy between the two platforms was tested to be significant (*p* < .001, Table 2).

### 3.3 Differences among Otu, Zotu, Dotu, and Cotu methods

The clean reads from Illumina Hiseq2500 and Ion Torrent S5 platforms were analyzed using four methods, Cotu, Dotu, Otu, and Zotu, and the outcomes are listed in Result [Link], [Link].zip (https://github.com/YanleiLiu1989/Cotu‐master). The reliabilities of these four methods were parameterized by sequence recovery, sequence length, and genetic distance.

#### 3.3.1 Sequence recovery

Next‐generation sequencing reads have random sequencing errors. Sequencing depth determines the accuracy of output sequences. We set a minimum depth of 10× for both platforms and a similarity of 97% for ITS and 99% for *matK* and *rbcL*. With these restrictions, the number of sequences recovered by the four methods varied slightly for the data from Illumina platform (Figure S9a) but remarkably for the data from Ion Torrent platform (Figure S9b). For the data from Illumina platform, all methods except Dotu recovered more than 350 (92.1%) sequences of five gene fragments of 380 samples. However, for the data from Ion Torrent platform, only Cotu recovered 350 sequences of four gene fragments. In general, Cotu recovered the highest number of sequences, whereas Dotu recovered the lowest number of sequences (Figure S9).

#### 3.3.2 Sequence length

Different sequence creation methods are based on different principles and use slightly different reads from the same sample, and therefore, the length of the sequences created by different methods varies. The sequences created by Cotu method were the longest for all DNA fragments (Table 3). The length differences of sequences created by different methods were tested significant in ANOVA (*p* < .01, Table 3).

**TABLE 3:** Statistical *F* test of length and accuracy of sequences created with four data processing methods using reads from Illumina HiSeq 2500 and Ion Torrent S5

	Illumina								Ion Torrent							
	Mean				*df*	MS	*F*	*p*	Mean				*df*	MS	*F*	*p*
	Cotu	Dotu	Otu	Zotu					Cotu	Dotu	Otu	Zotu				
Sequence length					3	3.153	262.36	0.000					2	7.005	444.68	0.000
ITS1	360.3	338.2	354.5	352.8					362.4		346.4	343.5				
ITS2	395.0	305.7	380.2	379.0					401.5		376.1	374.4				
matK1	338.8	338.7	332.2	332.2					338.4		332.7	329.3				
matK2	380.6	378.5	368.2	380.4					380.5		370.4	373.0				
rbcL1	375.2	347.4	374.5	375.2					377.0		361.5	357.3				
rbcL2	410.9	326.4	391.3	389.4					414.7		379.7	366.2				
Genetic distance					3	0.209	15.09	0.000					2	0.11	4.752	0.009
ITS1	0.024	0.037	0.035	0.034					0.084		0.138	0.139				
ITS2	0.020	0.030	0.023	0.023					0.065		0.070	0.071				
matK1	0.007	0.010	0.009	0.009					0.012		0.015	0.015				
matK2	0.006	0.007	0.007	0.010					0.012		0.018	0.018				
rbcL1	0.008	0.008	0.008	0.011					0.004		0.005	0.005				
rbcL2	0.003	0.010	0.011	0.010					0.003		0.004	0.004				
Standard deviation																
ITS1	0.090	0.117	0.114	0.111					0.163		0.261	0.262				
ITS2	0.078	0.115	0.078	0.077					0.165		0.161	0.161				
matK1	0.042	0.060	0.054	0.054					0.062		0.076	0.076				
matK2	0.033	0.036	0.036	0.039					0.059		0.089	0.089				
rbcL1	0.026	0.026	0.027	0.026					0.018		0.023	0.023				
rbcL2	0.017	0.039	0.047	0.045					0.009		0.018	0.018				

Note

Genetic distance is measured by genetic distances between created sequences and the reference sequence of the same sample. DADA2 was not applicable to the data from Ion Torrent platform due to extraordinary variability of sequence lengths.

Abbreviations: *df*, degree of freedom; *F*, value of F‐statistics; MS, mean square.

Standard deviation is calculated based on genetic distance.

#### 3.3.3 Genetic distance

The more accurate the sequences, the smaller the genetic distances between the output sequences and the reference sequences. Again, the sequences created by Cotu method were the most similar to the reference sequences with the smallest distances for all DNA fragments (Table 3). Likewise, the genetic distance difference of sequences was tested significant among the four methods (*p* < .01, Table 3).

## 4 DISCUSSION

It is believed that nearly two million species we know today are only a small fraction of total species diversity in the world (Mora et al., 2011). Nowadays, discovery of new species, especially microorganisms, is technique‐dependent. DNA (meta)barcoding is one of the most effective methods for species identification, and it has been used for evaluating species diversity (Chen et al., 2016), monitoring changes in microorganism composition in the environment (Barberán et al., 2015), identifying species in processed food or drug materials (Chin et al., 2016), etc. However, the DNA (meta)barcoding technology is heavily dependent on the species coverage of the reference library. Since the publication of the paper by Hebert et al. (2003), seventeen years have passed and very few such libraries have been constructed. In order to construct a reference library of high species coverage in a relatively short period of time, we have to overcome several major challenges.

### 4.1 The first challenge is high costs in raw data collections

A major investment for DNA barcode reference library construction is in DNA sequencing. Conventional Sanger sequencing is costly and of low efficiency. With minor technical modification in this study (Figure 2), different gene fragments of multiple samples can be sequenced simultaneously on NGS platforms, which significantly lowers sequencing costs. For example, a mixture containing 16 gene fragments of 384 samples can be sequenced in a sequencing library and data of 10G give an average sequencing depth of 3,255× theoretically. 10G NGS data cost less than $1,000, and the average cost of per final barcode sequence is about $0.15. Compared with about $2.8 cost of each Sanger sequence, the sequencing cost in NGS is only about 5% of that in the Sanger sequencing.

### 4.2 The second challenge is the perplexity in choosing NGS platform

Scientists are always trying to get more results and better done a research with less money. Wise selection of an NGS platform is crucial for obtaining high‐quality results and saving money. Base quality, read length, data sizes, sequencing depth, and cost efficiency should be taken into consideration when selecting an NGS platform. Owing to the relatively high base quality and low cost, Illumina sequencing platform and Ion torrent S5 platform are currently the most suitable platforms for conventional DNA barcoding compared with other sequencing platforms (PacBio, Nanopore, Sanger, and so on). For Illumina Hiseq PE250 and Ion Torrent S5 suitable for fragments of 400 bp, the former performs better than the latter in this study in terms of base quality (Figure S3), read length (Table 2), sequencing depth (Figure S2), and sequence accuracy (Table 3).

NGS platforms have been successfully used in DNA metabarcoding of microorganisms, such as bacteria and viruses (Krehenwinkel et al., 2019), but are not very commonly used for DNA barcode reference library construction. Both Illumina and Ion Torrent S5 platforms meet the requirement for conventional DNA barcodes in half or full length. Although the first few bases are prone to be wrongly sequenced and need to be treated with caution, the average genetic distances (0.014, 0.007, and 0.003 separately for ITS, *matK,* and *rbcL* output from Cotu) between the queries and the references are small enough, indicating the reliability of NGS platforms for conventional DNA barcoding.

### 4.3 The third challenge is the complexity of data processing

**FIGURE 3:**
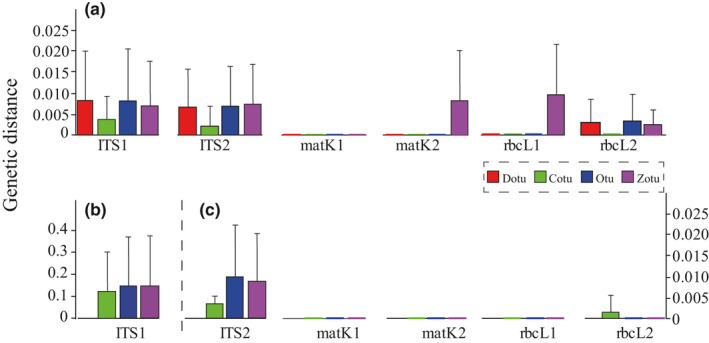
Accuracies of sequences created by Cotu (green), Dotu (red), Otu (blue), and Zotu (purple) methods with the data from Illumina Hiseq2500 (a) and Ion Torrent S5 (b & c) platforms. Average Kimura two‐parameter genetic distances between the queries and corresponding references were calculated with MEGA7. The Dotu method was not applicable to Ion Torrent S5 platform because read lengths were too variable to create reliable sequences. Ambiguous sites were not considered

The Cotu method has several advantages over other methods. (a) It separates contaminants by sorting the reads into groups in combination with Vsearch and creates sequences for every group. (b) It eliminates PCR and sequencing errors using consensus sequences under the majority rule. And (c) it maximizes lengths of output sequences using a user‐given threshold and avoids misuse of the majority rule. We compared this new method with other methods and found that the new method performed best in terms of both sequence length and sequence accuracy (Figure 3).

Unfortunately, as we have mentioned before, researchers have to face some software packages when doing different treatments of NGS data. In order to facilitate researchers who are not good at bioinformatics for Cotu generation, we packaged core Cotu steps together and named it “Cotu Master” (https://github.com/YanleiLiu1989/Cotu‐master) which is also provided in the Supporting [Link], [Link]. A few fastq datasets (Supporting data.zip) are provided for testing the program together with the expected results without (Result [Link], [Link]) or with 500 reads limit (Result [Link], [Link]) in https://github.com/YanleiLiu1989/Cotu‐master. Researchers can get their data just by entering a simple command following the step‐by‐step instructions (Supporting Document [Link], [Link], [Link]). Besides, in order to reduce the computing burden using Cotu method with an ordinary computer, an option of a maximum data usage was provided without lowering the quality of results. If the read number is larger than the maximum value (500×, for example), only the first 500 reads will be used.

### 4.4 The fourth challenge is the difficulties in determining thresholds

Most software packages are developed flexibly for users to input thresholds for NGS data processing. The similarity of reads to be grouped is one of the most important parameters to be determined before analyses. In the analyses of DNA metabarcoding data of microorganisms, a sequence similarity of 0.97 was often adopted for 16S, 18S, or COI fragments (Berry et al., 2017; Bremond et al., 2017; Yamamoto et al., 2017). The chloroplast plant DNA barcodes *matK* and *rbcL* are not so variable as nuclear barcode ITS (Figure S7 and Figure S8) and a different threshold of sequence similarity had better be used for situations of mixed samples or multiple gene copies. A sequence similarity of 0.99 is suitable for *matK* and *rbcL*, but lower similarity 0.97 may be appropriate for ITS. If the similarity value were set too high, the OTU diversity would be inflated. On the contrary, if the similarity value were set too low, differences between OTUs would be overwhelmed by the majority. For the Cotu method, no arbitrary similarity is necessary for reads of single copy fragments in a sample.

## 5 CONCLUSION

In order to support accurate molecular identification of organisms by means of DNA barcoding, a reference library with high species coverage needs to be constructed as quick and cheap as possible. To reach this goal, high‐throughput sequencing platforms are indispensable to speed up the processes and lower the costs. In this study, we show that the Illumina Hiseq PE250 is currently the right platform for conventional DNA barcodes. After comparing the newly developed data processing Cotu method to the existing Dotu, Otu, and Zotu methods, we conclude that the Cotu method is simpler, more accurate, and reliable. The packaged program Cotu master for creating consensus sequences had been uploaded to github (https://github.com/YanleiLiu1989/Cotu‐master). Besides, the user's manual (Supporting Document S1), step‐by‐step instructions (Supporting Document S2) are also provided for getting familiar to Cotu method more quickly. By using high‐throughput machines (PCR and NGS), labeling PCR, and the Cotu method, it is possible to significantly reduce the cost and labor investments for DNA barcoding. A regional or even global DNA barcoding reference library with high species coverage is likely to be constructed in a few years. As an example, a DNA reference library of seed plants in China is constructing using these methods and will soon be constructed with an investment of a few million dollars.

## CONFLICT OF INTEREST

The authors declare no conflict of interest.

## AUTHOR CONTRIBUTION

**Yanlei Liu:** Conceptualization (equal); Data curation (equal); Formal analysis (equal); Investigation (equal); Supervision (equal); Writing‐original draft (equal); Writing‐review & editing (equal). **Chao Xu:** Conceptualization (equal); Data curation (equal); Formal analysis (equal); Investigation (equal); Supervision (equal); Writing‐review & editing (equal). **Yuzhe Sun:** Formal analysis (equal); Writing‐review & editing (equal). **Xun Chen:** Formal analysis (equal); Investigation (equal). **Wenpan Dong:** Formal analysis (equal); Investigation (equal). **Xueying Yang:** Conceptualization (equal); Data curation (equal); Writing‐original draft (equal); Writing‐review & editing (equal). **Shi‐Liang Zhou:** Conceptualization (equal); Data curation (equal); Writing‐original draft (equal); Writing‐review & editing (equal).

## Supporting information

Fig S1

Fig S2

Fig S3

Fig S4

Fig S5

Fig S6

Fig S7

Fig S8

Fig S9

Table S1

Table S2

Supporting Document S1

Supporting Document S2

Data S1

Result S1

Result S2

Result S3

Software

## Data Availability

Sanger sequences of six gene fragments have been deposited in GenBank. The accession numbers are listed in Table S2. Original NGS data from Ion Torrent S5 and Illumina Hiseq2500 platforms have been submitted to NCBI with the accession numbers SRR11183118 and SRR11183119, respectively.
