# Supplementary material for: Method for quick DNA barcode reference library construction
Source: Ecol Evol. 2021 Aug 4;11(17):11627–38. doi: 10.1002/ece3.7788 (PMC8427591; doi:10.1002/ece3.7788)
Supplement: Supplementary file 1 — Fig S1 [file ECE3-11-11627-s001.pdf]

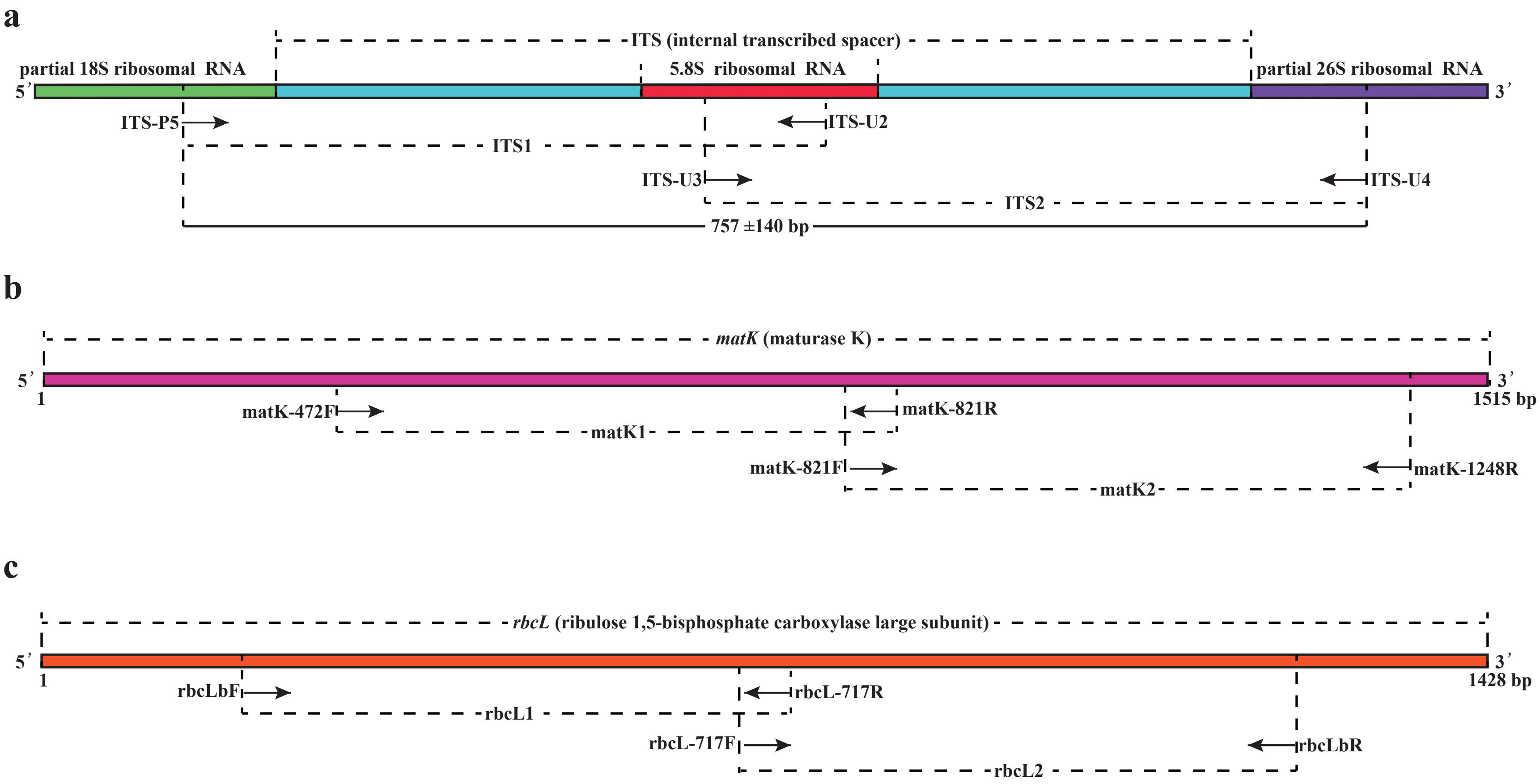

Fig. S1. Primers and their relative locations used in this study. a: Relative locations of forward and reverse primers used in ITS amplication; b: Relative locations of forward and reverse primers used in *matK* amplication; c: Relative locations of forward and reverse primers used in *rbcL* amplication. Right-pointing arrows represent forward primers while left-pointing arrows represent reverse primers.
