# Supplementary material for: Method for quick DNA barcode reference library construction
Source: Ecol Evol. 2021 Aug 4;11(17):11627–38. doi: 10.1002/ece3.7788 (PMC8427591; doi:10.1002/ece3.7788)
Supplement: Supplementary file 2 — Fig S2 [file ECE3-11-11627-s017.pdf]

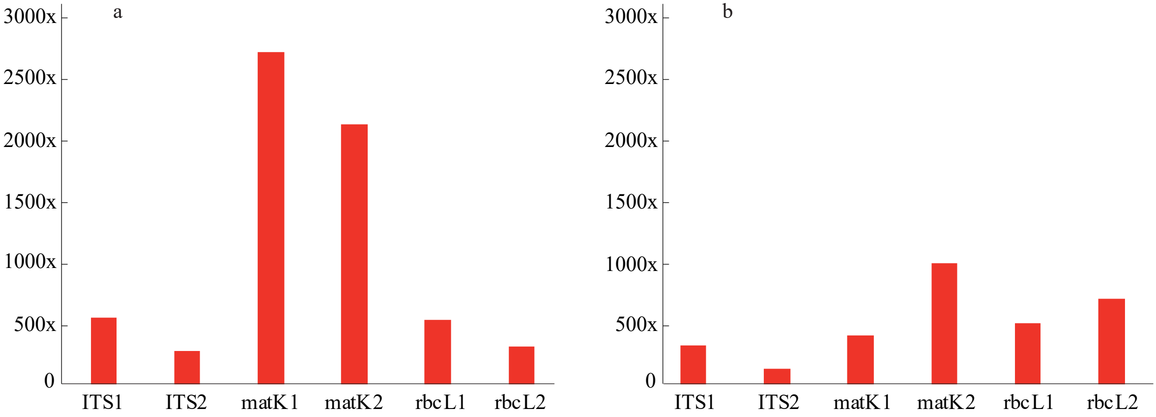

Fig. S2. Comparisons of average sequencing depths (vertical axis) of six gene fragments of 380 samples between Illumina Hiseq2500 (a) and Ion Torrent S5 (b) platforms.
