# Supplementary material for: Method for quick DNA barcode reference library construction
Source: Ecol Evol. 2021 Aug 4;11(17):11627–38. doi: 10.1002/ece3.7788 (PMC8427591; doi:10.1002/ece3.7788)
Supplement: Supplementary file 3 — Fig S3 [file ECE3-11-11627-s003.pdf]

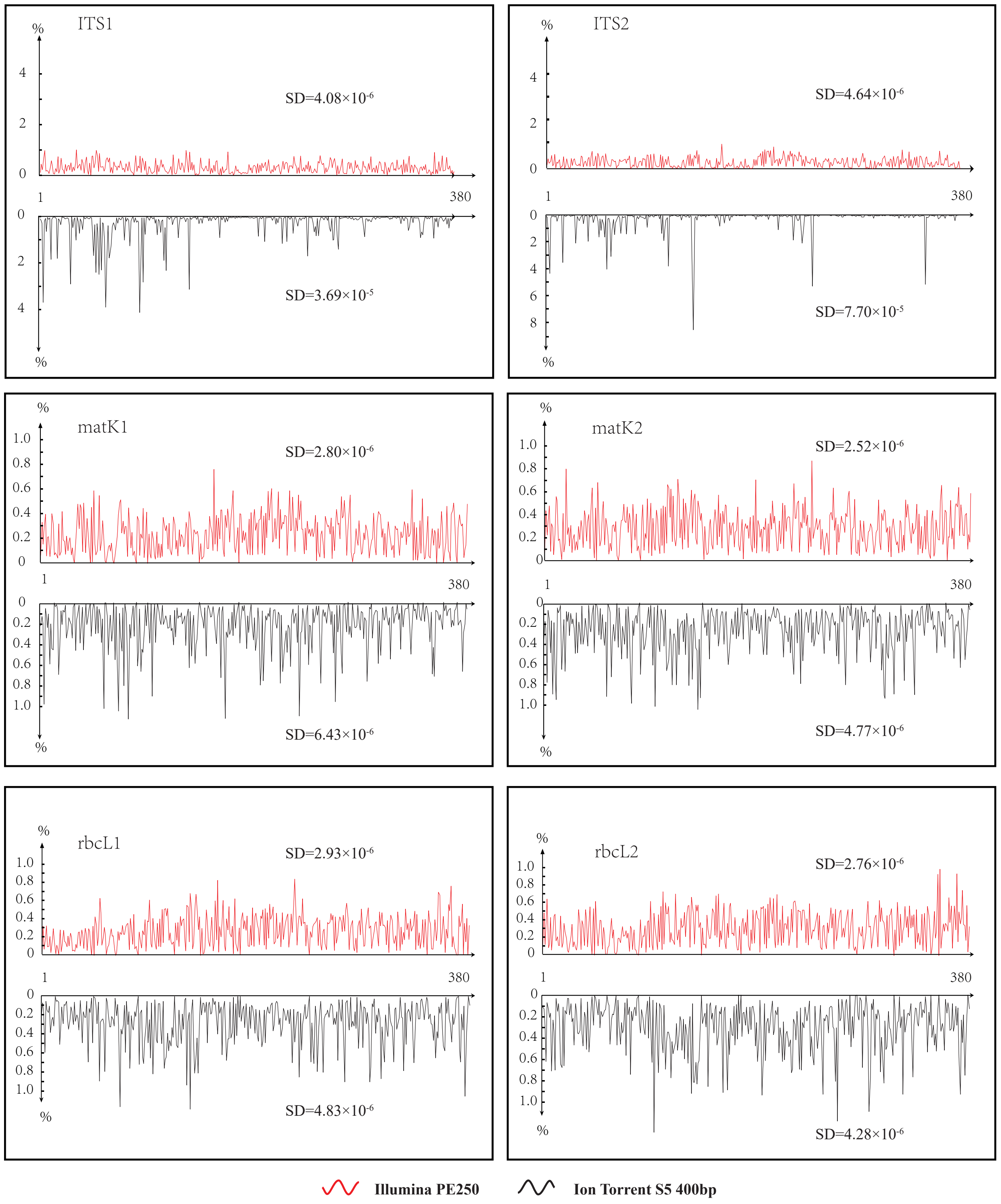

Fig. S3. Comparisons of average sequencing depth variations among samples between Illumina Hiseq2500 (red) and Ion Torrent S5 (blue) platforms. The horizontal axis represents the sample and the vertical axis represents the percentage of reads. SD is standard deviation.
