# Supplementary material for: Method for quick DNA barcode reference library construction
Source: Ecol Evol. 2021 Aug 4;11(17):11627–38. doi: 10.1002/ece3.7788 (PMC8427591; doi:10.1002/ece3.7788)
Supplement: Supplementary file 4 — Fig S4 [file ECE3-11-11627-s015.pdf]

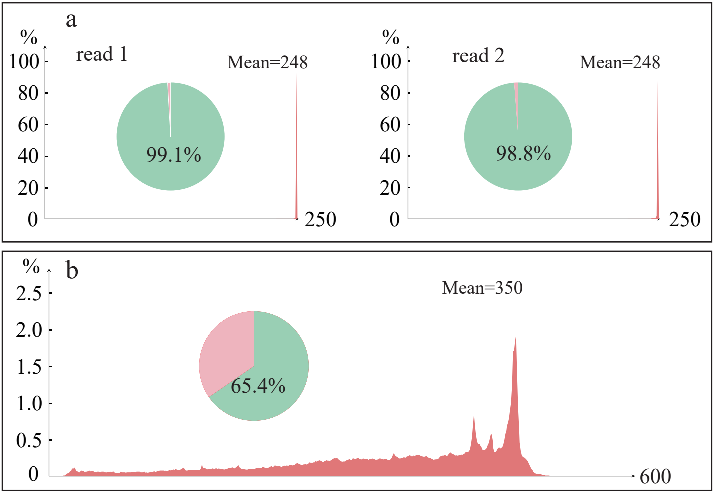

Fig. S4. Comparisons of average read length differences between Illumina Hiseq2500 and Ion Torrent S5 platforms. The horizontal axis is the read length and the vertical axis is the percentages to the total reads. The percentages in pie charts are the proportions of reads equal to or longer than 80% of the expected lengths, which are 200 bp for Illumina Hiseq2500 and 320 bp for Ion Torrent S5. a: Illumina Hiseq2500; and b: Ion Torrent S5.
