# Supplementary material for: Method for quick DNA barcode reference library construction
Source: Ecol Evol. 2021 Aug 4;11(17):11627–38. doi: 10.1002/ece3.7788 (PMC8427591; doi:10.1002/ece3.7788)
Supplement: Supplementary file 5 — Fig S5 [file ECE3-11-11627-s005.pdf]

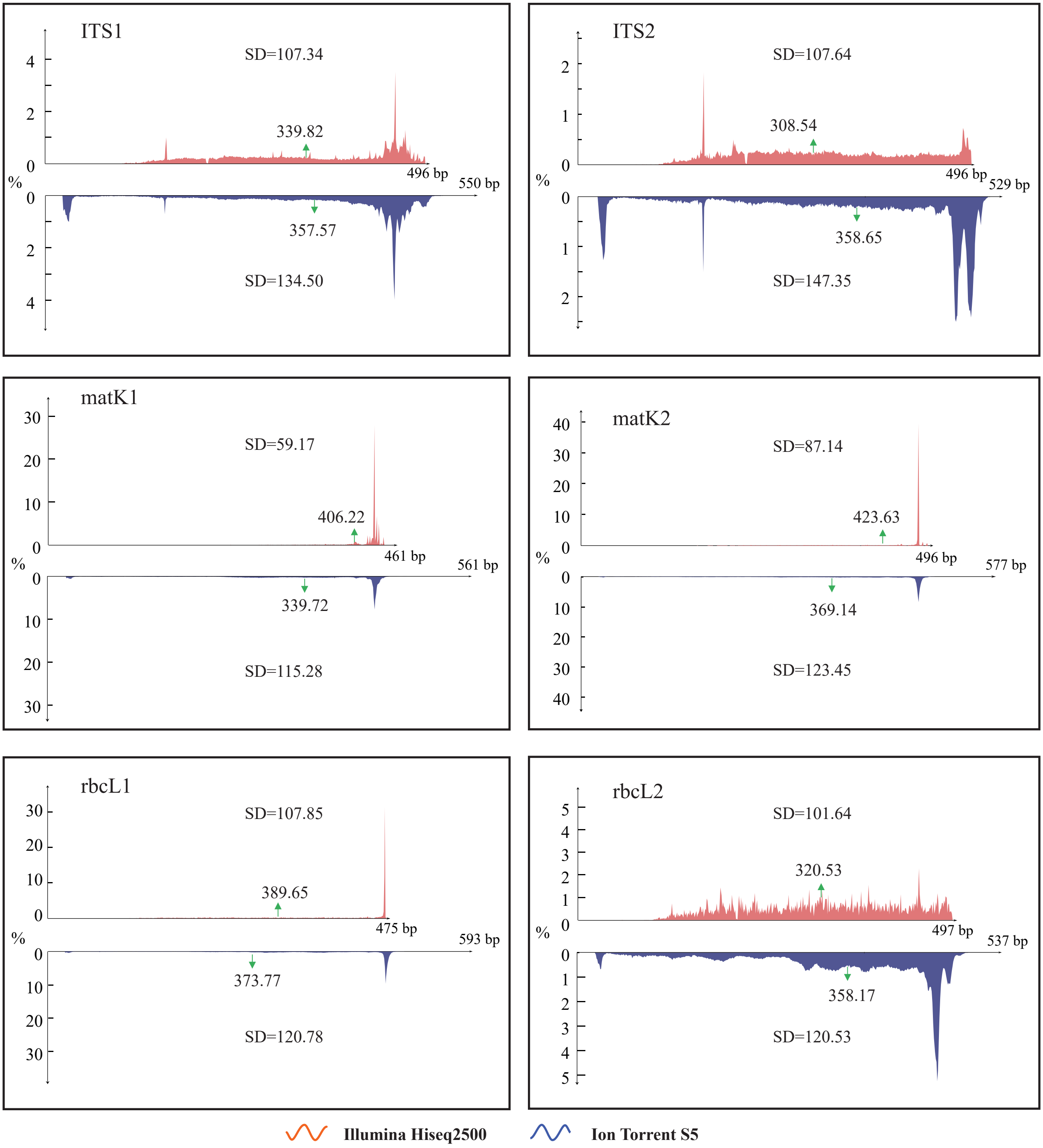

Fig. S5. Comparisons of average read length variations by gene fragments between Illumina Hiseq2500 (red) and Ion Torrent S5 (blue) platforms. The mean length values are positioned by green arrows. The horizontal axis is the read length and the vertical axis is the percentages of the reads.
