# Supplementary material for: Method for quick DNA barcode reference library construction
Source: Ecol Evol. 2021 Aug 4;11(17):11627–38. doi: 10.1002/ece3.7788 (PMC8427591; doi:10.1002/ece3.7788)
Supplement: Supplementary file 6 — Fig S6 [file ECE3-11-11627-s012.pdf]

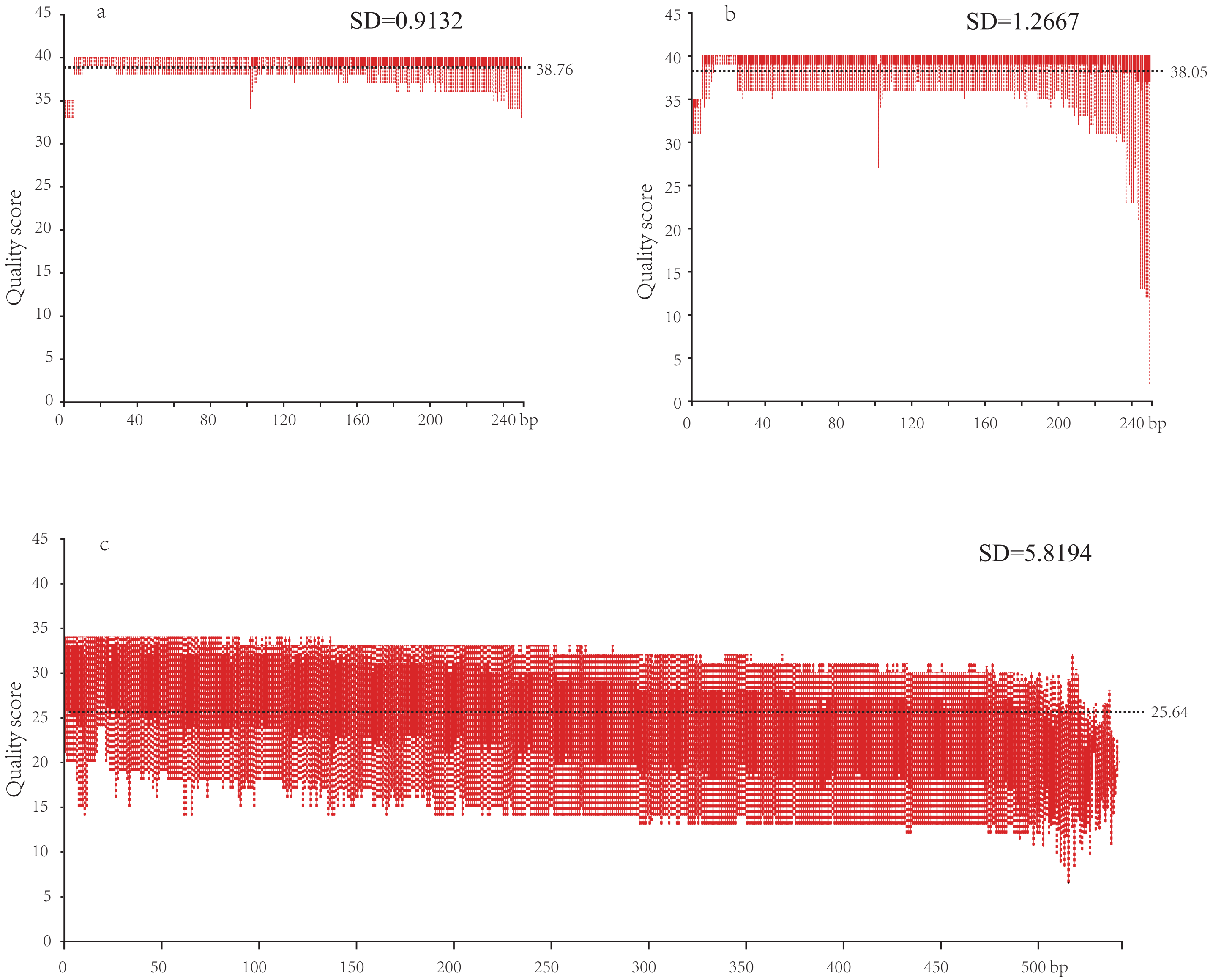

Fig. S6. Comparisons of average base quality differences between Illumina Hiseq2500 and Ion Torrent S5 platforms. The dotted line indicates the mean value.The horizontal axis is site position, the vertical axis is base quality score. a: Illumina Hiseq2500, read 1; b: Illumina Hiseq2500, read 2; and c: Ion Torrent S5.
