# Supplementary material for: Method for quick DNA barcode reference library construction
Source: Ecol Evol. 2021 Aug 4;11(17):11627–38. doi: 10.1002/ece3.7788 (PMC8427591; doi:10.1002/ece3.7788)
Supplement: Supplementary file 7 — Fig S7 [file ECE3-11-11627-s007.pdf]

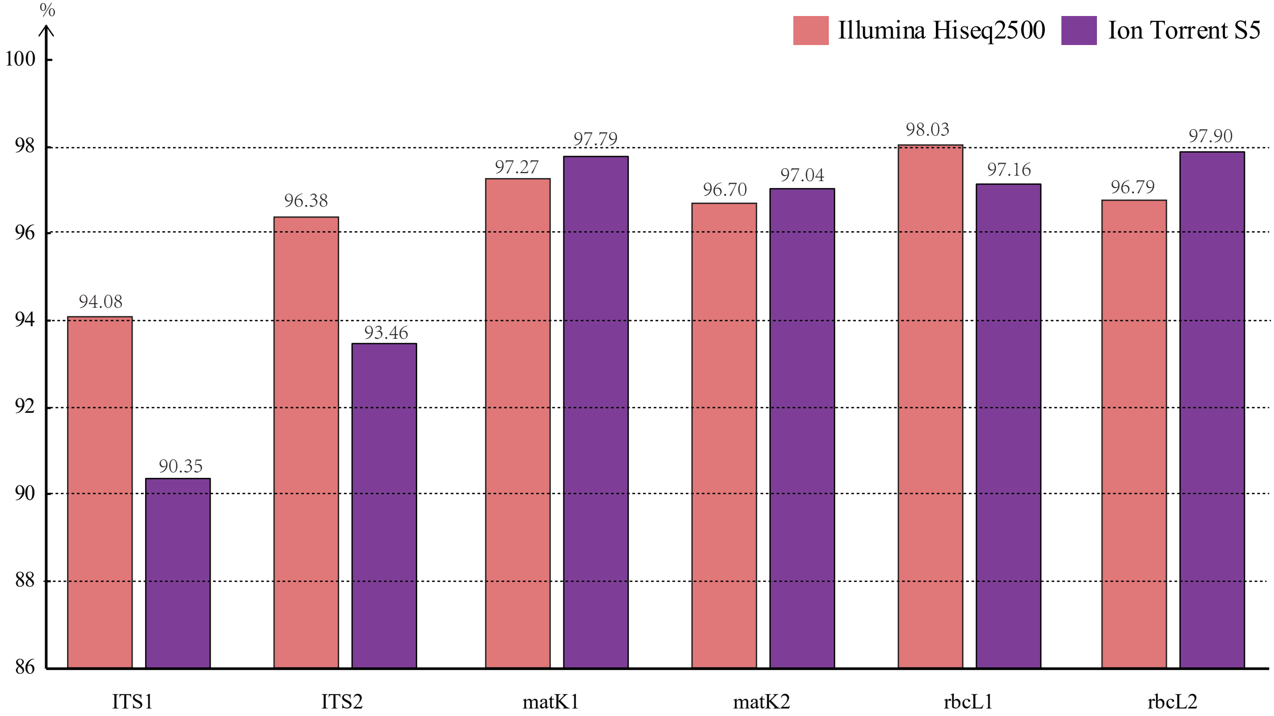

Fig. S7. Comparisons of average base accuracies by gene fragments between Illumina Hiseq2500 (red) and Ion Torrent S5 (purple) platforms. The vertical axis is percentage of correct bases to all bases.
