# Supplementary material for: Method for quick DNA barcode reference library construction
Source: Ecol Evol. 2021 Aug 4;11(17):11627–38. doi: 10.1002/ece3.7788 (PMC8427591; doi:10.1002/ece3.7788)
Supplement: Supplementary file 8 — Fig S8 [file ECE3-11-11627-s008.pdf]

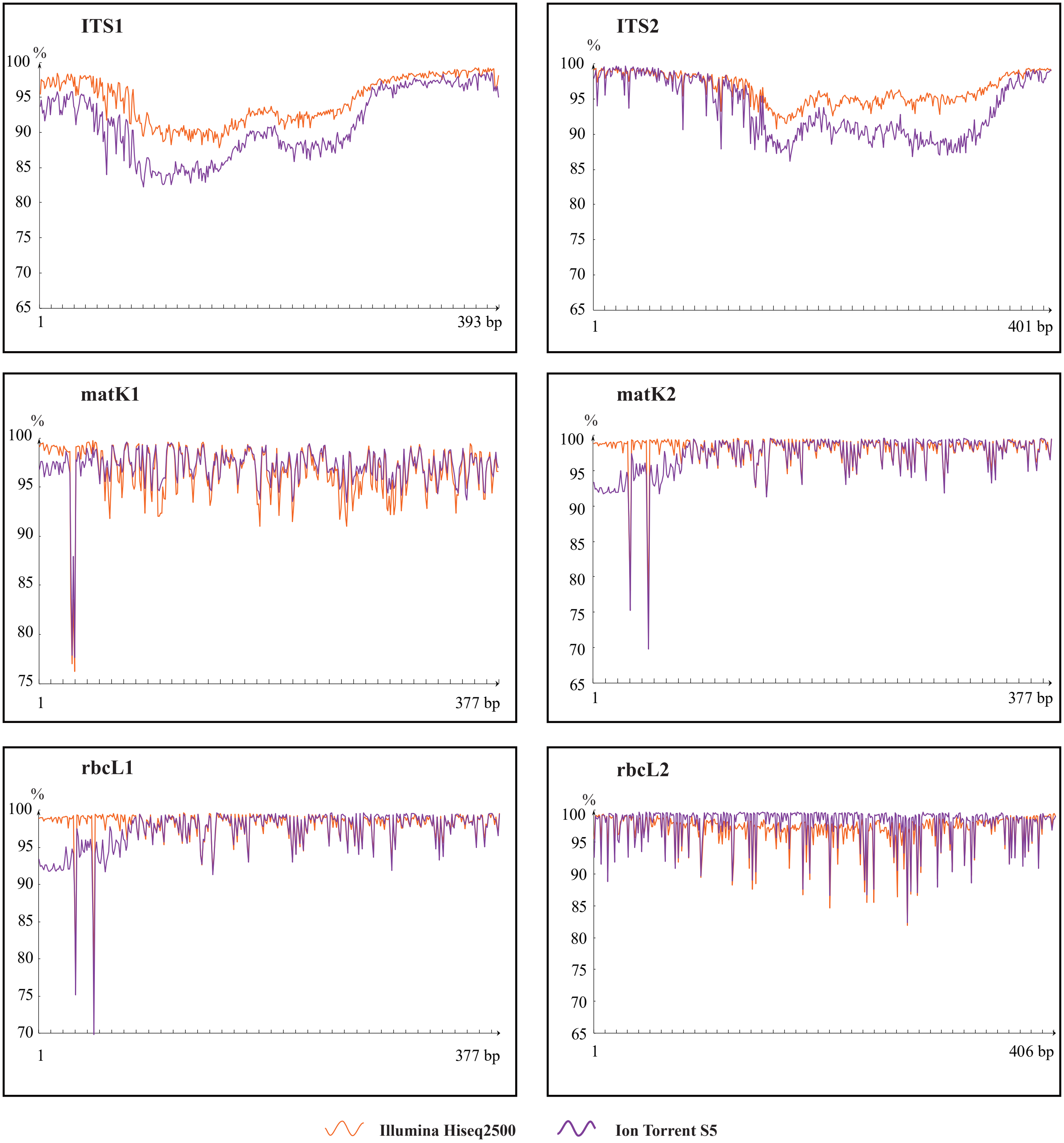

Fig. S8. Comparisons of average base accuracies by sites in six gene fragments between Illumina Hiseq2500 (red) and Ion Torrent S5 (purple) platforms. The horizontal axis is site position, the vertical axis is percentage of correct bases to all bases each site.
