# Supplementary material for: Method for quick DNA barcode reference library construction
Source: Ecol Evol. 2021 Aug 4;11(17):11627–38. doi: 10.1002/ece3.7788 (PMC8427591; doi:10.1002/ece3.7788)
Supplement: Supplementary file 9 — Fig S9 [file ECE3-11-11627-s011.pdf]

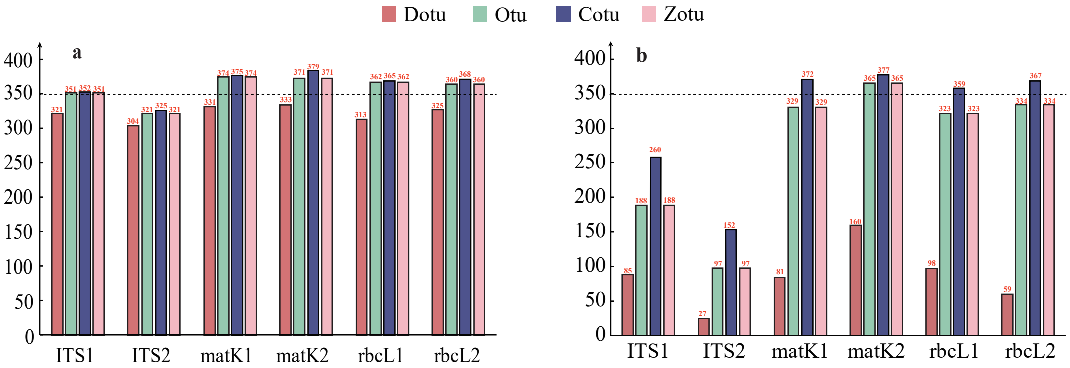

Fig. S9. Comparisons of the sequence quantitiess created by Cotu (purple), Dotu (red), Otu (green), and Zotu (pink) methods. a: Illumina Hiseq2500; b: Ion Torrent S5. The vertical axis represents the number of recovered sequences (380 in total).
