# Supplementary material for: Method for quick DNA barcode reference library construction
Source: Ecol Evol. 2021 Aug 4;11(17):11627–38. doi: 10.1002/ece3.7788 (PMC8427591; doi:10.1002/ece3.7788)
Supplement: Supplementary file 10 — Table S1 [file ECE3-11-11627-s013.docx]

**Table S1. Plant samples collected in Beijing Botanical Garden, Chinese Academy of Sciences and their DNA labels.**

| **Family** | **Genus** | **Species** | **Voucher** | **DNA label name** | **DNA label sequence (5'-3')** |
| --- | --- | --- | --- | --- | --- |
| Aceraceae | *Acer* | *davidii* | BOP010091 | barcode-072 | GCCTTGCCTT |
| Aceraceae | *Acer* | *ginnala* | BOP010199 | barcode-167 | GTATAGTATA |
| Aceraceae | *Acer* | *palmatum* | BOP010210 | barcode-178 | GAATAGAATA |
| Aceraceae | *Acer* | *pseudo-sieboldianum* | BOP010209 | barcode-177 | TACGGTACGG |
| Aceraceae | *Acer* | *semenovii* | BOP010201 | barcode-169 | TAACTTAACT |
| Aceraceae | *Acer* | sp. | BOP010198 | barcode-166 | CATAACATAA |
| Aceraceae | *Acer* | sp. | BOP010211 | barcode-179 | GCCGAGCCGA |
| Aceraceae | *Acer* | *stenolobum* | BOP010200 | barcode-168 | GGGATGGGAT |
| Aceraceae | *Acer* | *truncatum* | BOP010011 | barcode-009 | TTGTTTTGTT |
| Aceraceae | *Acer* | *truncatum* | BOP010195 | barcode-164 | TTATTTTATT |
| Aceraceae | *Acer* | sp. | BOP010207 | barcode-175 | AGGATAGGAT |
| Actinidiaceae | *Actinidia* | *chinensis* | BOP010142 | barcode-114 | TCACTTCACT |
| Actinidiaceae | *Actinidia* | sp. | BOP010141 | barcode-113 | AGGGAAGGGA |
| Actinidiaceae | *Actinidia* | sp. | BOP010479 | barcode-362 | AGCGAAGCGA |
| Alangiaceae | *Alangium* | *platanifolium* | BOP010311 | barcode-264 | GGGTCGGGTC |
| Anacardiaceae | *Cotinus* | *coggygria* | BOP010064 | barcode-046 | ATACAATACA |
| Anacardiaceae | *Pistacia* | *chinensis* | BOP010204 | barcode-172 | AAGATAAGAT |
| Anacardiaceae | *Rhus* | *chinensis* | BOP010160 | barcode-130 | ACTTAACTTA |
| Anacardiaceae | *Rhus* | *chinensis* | BOP010359 | barcode-301 | CGAGTCGAGT |
| Anacardiaceae | *Rhus* | *potaninii* | BOP010163 | barcode-133 | GTTTTGTTTT |
| Anacardiaceae | *Rhus* | *punjabensis var.sinica* | BOP010074 | barcode-056 | ACTCGACTCG |
| Anacardiaceae | *Rhus* | *typhina* | BOP010203 | barcode-171 | AGGGTAGGGT |
| Anacardiaceae | *Toxicodendron* | *delavayi* | BOP010202 | barcode-170 | CCATACCATA |
| Araliaceae | *Aralia* | *chinensis* | BOP010322 | barcode-274 | GCGCCGCGCC |
| Araliaceae | *Eleutherococcus* | *gracilistylus* | BOP010323 | barcode-275 | CCTCACCTCA |
| Araliaceae | *Eleutherococcus* | *sessiflorus* | BOP010019 | barcode-016 | TCTCTTCTCT |
| Araliaceae | *Kalopanax* | *septemlobus* | BOP010325 | barcode-277 | TTCTCTTCTC |
| Araliaceae | *Tetrapanax* | *papyrifer* | BOP010454 | barcode-340 | GACCGGACCG |
| Aristolochiaceae | *Aristolochia* | *manshuriensis* | BOP010101 | barcode-082 | GGAGAGGAGA |
| Aristolochiaceae | *Callicarpa* | *americana* | BOP010293 | barcode-248 | CCGCGCCGCG |
| Aristolochiaceae | *Callicarpa* | *dichotoma* | BOP010295 | barcode-250 | CTTAACTTAA |
| Aristolochiaceae | *Callicarpa* | sp. | BOP010135 | barcode-107 | TCAAGTCAAG |
| Berberidaceae | *Berberis* | *brachypoda* | BOP010078 | barcode-060 | TACCGTACCG |
| Berberidaceae | *Berberis* | *brachypoda* | BOP010082 | barcode-064 | AACTGAACTG |
| Berberidaceae | *Berberis* | *diaphana* | BOP010069 | barcode-051 | GCGTAGCGTA |
| Berberidaceae | *Berberis* | sp. | BOP010067 | barcode-049 | ACCACACCAC |
| Berberidaceae | *Berberis* | sp. | BOP010070 | barcode-052 | ATATAATATA |
| Berberidaceae | *Berberis* | sp. | BOP010073 | barcode-055 | GTAAGGTAAG |
| Berberidaceae | *Berberis* | sp. | BOP010076 | barcode-058 | TAGCCTAGCC |
| Berberidaceae | *Berberis* | sp. | BOP010077 | barcode-059 | CCGCCCCGCC |
| Berberidaceae | *Berberis* | sp. | BOP010079 | barcode-061 | CGACACGACA |
| Berberidaceae | *Berberis* | sp. | BOP010080 | barcode-062 | ACACAACACA |
| Berberidaceae | *Berberis* | sp. | BOP010081 | barcode-063 | ACGGAACGGA |
| Berberidaceae | *Berberis* | sp. | BOP010083 | barcode-065 | TTATATTATA |
| Berberidaceae | *Berberis* | sp. | BOP010084 | barcode-066 | CCTTACCTTA |
| Berberidaceae | *Berberis* | *thunbergii* | BOP010068 | barcode-050 | TGGATTGGAT |
| Berberidaceae | *Berberis* | *thunbergii* | BOP010136 | barcode-108 | GGAAGGGAAG |
| Berberidaceae | *Nandina* | *domestica* | BOP010491 | barcode-370 | TGGAGTGGAG |
| Betulaceae | *Betula* | *alnoides* | BOP010014 | barcode-012 | AGATGAGATG |
| Betulaceae | *Carpinus* | sp. | BOP010436 | barcode-323 | AGATCAGATC |
| Betulaceae | *Carpinus* | *turczaninowii* | BOP010434 | barcode-321 | CGCATCGCAT |
| Betulaceae | *Corylus* | *heterophylla* | BOP010330 | barcode-281 | ATCTAATCTA |
| Bignoniaceae | *Campsis* | *grandiflora* | BOP010266 | barcode-225 | AAACCAAACC |
| Bignoniaceae | *Catalpa* | *ovata* | BOP010238 | barcode-202 | CTGCGCTGCG |
| Bignoniaceae | *Catalpa* | *ovata* | BOP010458 | barcode-343 | GCTTTGCTTT |
| Bignoniaceae | *Catalpa* | sp. | BOP010227 | barcode-191 | ATACTATACT |
| Buxaceae | *Buxus* | *sinica var.parvifolia* | BOP010025 | barcode-020 | AAATGAAATG |
| Buxaceae | *Buxus* | *sinica_var.parvifolia* | BOP010223 | barcode-188 | GGCCCGGCCC |
| Buxaceae | *Pachysandra* | *terminalis* | BOP010463 | barcode-348 | CCCTGCCCTG |
| Calycanthaceae | *Calycanthus* | *chinensis* | BOP010108 | barcode-088 | GCTTAGCTTA |
| Campanulaceae | *Campanula* | *punctata* | BOP010286 | barcode-242 | CAGTACAGTA |
| Caprifoliaceae | *Abelia* | *chinensis* | BOP010416 | barcode-303 | ATGTTATGTT |
| Caprifoliaceae | *Lonicera* | *fragrantissima* | BOP010122 | barcode-098 | TCCTTTCCTT |
| Caprifoliaceae | *Lonicera* | *fragrantissima* | BOP010287 | barcode-243 | GGGTGGGGTG |
| Caprifoliaceae | *Lonicera* | *japonica* | BOP010152 | barcode-122 | CAACCCAACC |
| Caprifoliaceae | *Lonicera* | *japonica* | BOP010449 | barcode-336 | TCTACTCTAC |
| Caprifoliaceae | *Lonicera* | *maackii* | BOP010053 | barcode-036 | AGGAGAGGAG |
| Caprifoliaceae | *Lonicera* | *microphylla* | BOP010291 | barcode-246 | TCCATTCCAT |
| Caprifoliaceae | *Lonicera* | sp. | BOP010288 | barcode-244 | GAGTAGAGTA |
| Caprifoliaceae | *Lonicera* | *tatarica* | BOP010281 | barcode-239 | CTAGTCTAGT |
| Caprifoliaceae | *Lonicera* | *tatarica* | BOP010283 | barcode-241 | CCCGTCCCGT |
| Caprifoliaceae | *Lonicera* | *tatarica* | BOP010292 | barcode-247 | GCAAGGCAAG |
| Caprifoliaceae | *Lonicera* | *tellmanniana* | BOP010448 | barcode-335 | CCCGACCCGA |
| Caprifoliaceae | *Loricera* | *korolkowii* | BOP010294 | barcode-249 | TTATGTTATG |
| Caprifoliaceae | *Sambucus* | *canaclensis* | BOP010269 | barcode-227 | TCCTCTCCTC |
| Caprifoliaceae | *Viburnum* | *burejaeticum* | BOP010262 | barcode-221 | CGCCACGCCA |
| Caprifoliaceae | *Viburnum* | *carlesii* | BOP010257 | barcode-217 | CGTTCCGTTC |
| Caprifoliaceae | *Viburnum* | *dentatum* | BOP010259 | barcode-219 | GAGTTGAGTT |
| Caprifoliaceae | *Viburnum* | *dilatatum* | BOP010158 | barcode-128 | TAGCTTAGCT |
| Caprifoliaceae | *Viburnum* | *farreri* | BOP010166 | barcode-135 | GTAATGTAAT |
| Caprifoliaceae | *Viburnum* | *melanocarpum* | BOP010260 | barcode-220 | TTACTTTACT |
| Caprifoliaceae | *Viburnum* | *opulus_var.calvescens* | BOP010245 | barcode-208 | CCGGACCGGA |
| Caprifoliaceae | *Viburnum* | *prunifolium* | BOP010254 | barcode-215 | CCACCCCACC |
| Caprifoliaceae | *Viburnum* | *rhytidophyllum* | BOP010258 | barcode-218 | GACTAGACTA |
| Caprifoliaceae | *Viburnum* | sp. | BOP010246 | barcode-209 | GTCGCGTCGC |
| Caprifoliaceae | *Viburnum* | sp. | BOP010247 | barcode-210 | ACGATACGAT |
| Caprifoliaceae | *Viburnum* | sp. | BOP010255 | barcode-216 | CCCATCCCAT |
| Caprifoliaceae | *Weigela* | *florida* | BOP010230 | barcode-194 | TCAGTTCAGT |
| Caprifoliaceae | *Weigela* | *florida* | BOP010248 | barcode-211 | CGGAGCGGAG |
| Caprifoliaceae | *Zabelia* | *biflora* | BOP010125 | barcode-099 | TCGGTTCGGT |
| Casuarinaceae | *Casuarina* | *equisetifolia* | BOP010492 | barcode-371 | ATGAGATGAG |
| Celastraceae | *Euongmus* | *japonica* | BOP010131 | barcode-104 | CACGTCACGT |
| Celastraceae | *Euongmus* | *kiautschovicus* | BOP010252 | barcode-214 | AGAACAGAAC |
| Celastraceae | *Euonymus* | *alatus* | BOP010126 | barcode-100 | CACAACACAA |
| Celastraceae | *Euonymus* | *bungeanus* | BOP010129 | barcode-103 | TTGTATTGTA |
| Celastraceae | *Euonymus* | *fortunei* | BOP010087 | barcode-069 | CCATCCCATC |
| Celastraceae | *Euonymus* | sp. | BOP010127 | barcode-101 | CCACTCCACT |
| Celastraceae | *Euonymus* | sp. | BOP010128 | barcode-102 | TGCGCTGCGC |
| Cornaceae | *Cornus* | *alba* | BOP010093 | barcode-074 | GCCCCGCCCC |
| Cornaceae | *Cornus* | *walteri* | BOP010312 | barcode-265 | GGAGTGGAGT |
| Cornaceae | *Cornus* | *walteri* | BOP010313 | barcode-266 | GATGAGATGA |
| Cornaceae | *Cornus* | *walteri* | BOP010446 | barcode-333 | ATCGGATCGG |
| Cornaceae | *Dendrobenthamia* | *japonica var.chinensis* | BOP010164 | barcode-134 | GCACTGCACT |
| Cornaceae | *Dendrobenthamia* | *japonica_var.chinensis* | BOP010315 | barcode-268 | ATTATATTAT |
| Cornaceae | *Dendrobenthamia* | *japonica_var.chinensis* | BOP010316 | barcode-269 | CGTAACGTAA |
| Cornaceae | *Macrocarpium* | *officinalis* | BOP010048 | barcode-031 | CAGTGCAGTG |
| Ebenaceae | *Diospyros* | *cathayensis* | BOP010150 | barcode-120 | CACCCCACCC |
| Ebenaceae | *Diospyros* | *cathayensis* | BOP010151 | barcode-121 | TGATGTGATG |
| Ebenaceae | *Diospyros* | *cathayensis* | BOP010244 | barcode-207 | TAACGTAACG |
| Ebenaceae | *Diospyros* | *lotus* | BOP010186 | barcode-155 | AAGCTAAGCT |
| Ebenaceae | *Diospyros* | *virginiana* | BOP010243 | barcode-206 | GATTGGATTG |
| Elaeagnaceae | *Elaeagnus* | *angustifolia* | BOP010326 | barcode-278 | TCGTATCGTA |
| Elaeagnaceae | *Elaeagnus* | sp. | BOP010119 | barcode-095 | AGTCTAGTCT |
| Elaeagnaceae | *Elaeagnus* | *umbellata* | BOP010319 | barcode-272 | GTGCAGTGCA |
| Elaeagnaceae | *Elaeagnus* | *umbellata* | BOP010320 | barcode-273 | TTAAGTTAAG |
| Euphorbiaceae | *Flueggea* | *suffruticosa* | BOP010052 | barcode-035 | GCCCTGCCCT |
| Fabaceae | *Albizia* | *julibrissin* | BOP010324 | barcode-276 | AGCTCAGCTC |
| Fabaceae | *Amorpha* | *fruticosa* | BOP010050 | barcode-033 | GGATGGGATG |
| Fabaceae | *Caragana* | *frutex* | BOP010063 | barcode-045 | CCGGTCCGGT |
| Fabaceae | *Cercis* | *canadensis* | BOP010060 | barcode-042 | ACATAACATA |
| Fabaceae | *Cercis* | *chinensis* | BOP010459 | barcode-344 | ACTGCACTGC |
| Fabaceae | *Cercis* | *gigantea* | BOP010059 | barcode-041 | TCGGCTCGGC |
| Fabaceae | *Coluten* | *arborescens* | BOP010270 | barcode-228 | AAGTGAAGTG |
| Fabaceae | *Gleditsia* | *sinensis* | BOP010054 | barcode-037 | CCTTCCCTTC |
| Fabaceae | *Gleditsia* | *triacanthos* | BOP010444 | barcode-331 | AATAAAATAA |
| Fabaceae | *Halimodendron* | *halodendron* | BOP010290 | barcode-245 | ATGTAATGTA |
| Fabaceae | *Indigofera* | *kirilowii* | BOP010061 | barcode-043 | AACCTAACCT |
| Fabaceae | *Pueraria* | sp. | BOP010161 | barcode-131 | GGGAAGGGAA |
| Fabaceae | *Sophora* | *davidii* | BOP010062 | barcode-044 | TAAGATAAGA |
| Fabaceae | *Sophora* | *japonica* | BOP010088 | barcode-070 | CTAGACTAGA |
| Fabaceae | *Sophora* | *viciifolia* | BOP010272 | barcode-230 | AAGTCAAGTC |
| Fabaceae | *Wisteria* | *floribunda* | BOP010102 | barcode-083 | TTGTCTTGTC |
| Fabaceae | *Caragana* | sp. | BOP010168 | barcode-137 | AGGAAAGGAA |
| Fabaceae | *Caragana* | sp. | BOP010469 | barcode-353 | TCCGGTCCGG |
| Fagaceae | *Castanea* | *mollissima* | BOP010328 | barcode-279 | CAACTCAACT |
| Fagaceae | *Quercus* | *aliena* | BOP010423 | barcode-310 | AAAGTAAAGT |
| Fagaceae | *Quercus* | *aliena* | BOP010439 | barcode-326 | TGGCTTGGCT |
| Fagaceae | *Quercus* | *aliena_var.acuteserrata* | BOP010421 | barcode-308 | TGTGATGTGA |
| Fagaceae | *Quercus* | *aliena_var.acuteserrata* | BOP010422 | barcode-309 | CTTCCCTTCC |
| Fagaceae | *Quercus* | *aliena_var.acuteserrata* | BOP010442 | barcode-329 | ACAAGACAAG |
| Fagaceae | *Quercus* | *baronii* | BOP010441 | barcode-328 | GCGAAGCGAA |
| Fagaceae | *Quercus* | *dentata* | BOP010271 | barcode-229 | ATCTGATCTG |
| Fagaceae | *Quercus* | *fabri* | BOP010420 | barcode-307 | CCTGTCCTGT |
| Fagaceae | *Quercus* | *fabri* | BOP010426 | barcode-313 | CCGACCCGAC |
| Fagaceae | *Quercus* | *gambelii* | BOP010431 | barcode-318 | GGCGTGGCGT |
| Fagaceae | *Quercus* | *glandulifera_var.brevipetiolata* | BOP010427 | barcode-314 | ACGTGACGTG |
| Fagaceae | *Quercus* | *macrocalyx* | BOP010443 | barcode-330 | CAAGGCAAGG |
| Fagaceae | *Quercus* | *macrocarpa* | BOP010428 | barcode-315 | TCGTTTCGTT |
| Fagaceae | *Quercus* | *palustris* | BOP010429 | barcode-316 | AAGTAAAGTA |
| Fagaceae | *Quercus* | *robur* | BOP010418 | barcode-305 | CGGCTCGGCT |
| Fagaceae | *Quercus* | *robur* | BOP010419 | barcode-306 | TCAATTCAAT |
| Fagaceae | *Quercus* | *rubra* | BOP010433 | barcode-320 | TATGTTATGT |
| Fagaceae | *Quercus* | *serrata* | BOP010440 | barcode-327 | AGTACAGTAC |
| Fagaceae | *Quercus* | sp. | BOP010417 | barcode-304 | CGTATCGTAT |
| Fagaceae | *Quercus* | sp. | BOP010425 | barcode-312 | CCAAACCAAA |
| Fagaceae | *Quercus* | sp. | BOP010430 | barcode-317 | CGGGACGGGA |
| Fagaceae | *Quercus* | *stellata* | BOP010424 | barcode-311 | ATCGAATCGA |
| Fagaceae | *Quercus* | *variabilis* | BOP010445 | barcode-332 | TCGTGTCGTG |
| Hippocastanaceae | *Aesculus* | *chinensis* | BOP010140 | barcode-112 | ACTTGACTTG |
| Hippocastanaceae | *Aesculus* | *chinensis* | BOP010250 | barcode-213 | TGCTATGCTA |
| Hippocastanaceae | *Aesculus* | sp. | BOP010155 | barcode-125 | GTAGTGTAGT |
| Hippocastanaceae | *Aesculus* | *turbinata* | BOP010249 | barcode-212 | CCCACCCCAC |
| Hydrangeaceae | *Deutzia* | *parviflora* | BOP010045 | barcode-029 | TGATTTGATT |
| Hydrangeaceae | *Hydrangea* | *macrophylla_f.otaksa* | BOP010452 | barcode-338 | AATGCAATGC |
| Hydrangeaceae | *Philadelphus* | *coronarius* | BOP010219 | barcode-184 | CCGTTCCGTT |
| Hydrangeaceae | *Philadelphus* | *lemoiner* | BOP010218 | barcode-183 | ATCGCATCGC |
| Hydrangeaceae | *Philadelphus* | *pekinensis* | BOP010046 | barcode-030 | TGTGGTGTGG |
| Hydrangeaceae | *Philadelphus* | *pekinensis* | BOP010220 | barcode-185 | TAGAGTAGAG |
| Hydrangeaceae | *Philadelphus* | *pekinensis* | BOP010221 | barcode-186 | CAGCTCAGCT |
| Hydrangeaceae | *Philadelphus* | *pekinensis* | BOP010222 | barcode-187 | TGTGTTGTGT |
| Hydrangeaceae | *Philadelphus* | *pekinensis* | BOP010465 | barcode-350 | TGCATTGCAT |
| Juglandaceae | *Carya* | *cathayensis* | BOP010299 | barcode-253 | TTGCATTGCA |
| Juglandaceae | *Juglans* | *mandshurica* | BOP010116 | barcode-092 | GAAATGAAAT |
| Juglandaceae | *Juglans* | *regia* | BOP010117 | barcode-093 | TTGAGTTGAG |
| Juglandaceae | *Juglans* | sp. | BOP010348 | barcode-297 | AAGAGAAGAG |
| Juglandaceae | *Pterocarya* | sp. | BOP010297 | barcode-252 | TGCCTTGCCT |
| Juglandaceae | *Pterocarya* | *stenoptera* | BOP010296 | barcode-251 | ACTGAACTGA |
| Lamiaceae | *Elsholtzia* | *stauntoni* | BOP010179 | barcode-148 | CGATCCGATC |
| Lauraceae | *Laurus* | *nobilis* | BOP010497 | barcode-376 | CGTTTCGTTT |
| Lauraceae | *Lindera* | *glauca* | BOP010154 | barcode-124 | AACACAACAC |
| Loganiaceae | *Buddleja* | *albiflora* | BOP010282 | barcode-240 | CGCTCCGCTC |
| Lythraceae | *Lagerstroemia* | *indica* | BOP010171 | barcode-140 | CGAATCGAAT |
| Magnoliaceae | *Liriodendron* | *chinense* | BOP010099 | barcode-080 | GCCTGGCCTG |
| Magnoliaceae | *Magnolia* | *amoena* | BOP010098 | barcode-079 | AGCTTAGCTT |
| Magnoliaceae | *Magnolia* | *bionlii* | BOP010156 | barcode-126 | CCTGCCCTGC |
| Magnoliaceae | *Magnolia* | *cylindrica* | BOP010104 | barcode-084 | TACTGTACTG |
| Magnoliaceae | *Magnolia* | *soulangeana* | BOP010455 | barcode-341 | GCGGGGCGGG |
| Magnoliaceae | *Magnolia* | sp. | BOP010092 | barcode-073 | ACCTCACCTC |
| Magnoliaceae | *Magnolia* | *zenii* | BOP010100 | barcode-081 | TGACGTGACG |
| Malvaceae | *Grewia* | *biloba* | BOP010476 | barcode-359 | GCGGAGCGGA |
| Malvaceae | *Hibiscus* | *syriacus* | BOP010090 | barcode-071 | CCCAGCCCAG |
| Meliaceae | *Melia* | *azedarach* | BOP010460 | barcode-345 | CACCGCACCG |
| Meliaceae | *Toona* | *sinensis* | BOP010473 | barcode-357 | CTCTCCTCTC |
| Moraceae | *Broussonetia* | *papyifera* | BOP010317 | barcode-270 | GGGCTGGGCT |
| Moraceae | *Ficus* | *carica* | BOP010474 | barcode-358 | CTGACCTGAC |
| Moraceae | *Ficus* | *religiosa* | BOP010493 | barcode-372 | CTTGACTTGA |
| Moraceae | *Maclura* | *pomifera* | BOP010191 | barcode-160 | CCGGCCCGGC |
| Moraceae | *Maclura* | *tricuspidata* | BOP010167 | barcode-136 | TCATTTCATT |
| Moraceae | *Morus* | *alba* | BOP010318 | barcode-271 | GGGCCGGGCC |
| Moraceae | *Humulus* | *lupulus* | BOP010139 | barcode-111 | AATATAATAT |
| Musaceae | *Musa* | sp. | BOP010490 | barcode-369 | GAGACGAGAC |
| Myrtaceae | *Syzggiam* | *samaragense* | BOP010488 | barcode-368 | ACAGTACAGT |
| Oleaceae | *Chionanthus* | *retusus* | BOP010231 | barcode-195 | AATCCAATCC |
| Oleaceae | *Fontanesia* | *fortunei* | BOP010121 | barcode-097 | GGAACGGAAC |
| Oleaceae | *Fontanesia* | *phillyreoides subspfortunei* | BOP010056 | barcode-039 | GCCTAGCCTA |
| Oleaceae | *Forsythia* | *ovata* | BOP010275 | barcode-233 | AATGGAATGG |
| Oleaceae | *Forsythia* | *suspensa* | BOP010094 | barcode-075 | ATTCAATTCA |
| Oleaceae | *Forsythia* | *suspensa* | BOP010274 | barcode-232 | GAACGGAACG |
| Oleaceae | *Forsythia* | *viridissima* | BOP010273 | barcode-231 | CAATCCAATC |
| Oleaceae | *Fraxinus* | *americana* | BOP010237 | barcode-201 | GAGTCGAGTC |
| Oleaceae | *Fraxinus* | *baroniana* | BOP010224 | barcode-189 | TTCACTTCAC |
| Oleaceae | *Fraxinus* | *bungeana* | BOP010229 | barcode-193 | CTCCACTCCA |
| Oleaceae | *Fraxinus* | *excelsior_var.aurea* | BOP010235 | barcode-199 | GTAAAGTAAA |
| Oleaceae | *Fraxinus* | *hubeiensis* | BOP010236 | barcode-200 | TCCTATCCTA |
| Oleaceae | *Fraxinus* | *mandshurica* | BOP010120 | barcode-096 | GCGGTGCGGT |
| Oleaceae | *Fraxinus* | *paxiana* | BOP010085 | barcode-067 | TTCTTTTCTT |
| Oleaceae | *Fraxinus* | *pennsylvanica* | BOP010233 | barcode-197 | AGTAAAGTAA |
| Oleaceae | *Fraxinus* | *pennsylvanica_var.subintegerrima* | BOP010241 | barcode-204 | GCACAGCACA |
| Oleaceae | *Fraxinus* | *rhynchophylla* | BOP010232 | barcode-196 | ATTCTATTCT |
| Oleaceae | *Fraxinus* | sp. | BOP010228 | barcode-192 | CGTCACGTCA |
| Oleaceae | *Fraxinus* | sp. | BOP010234 | barcode-198 | TGTACTGTAC |
| Oleaceae | *Fraxinus* | *velutina* | BOP010226 | barcode-190 | AACAGAACAG |
| Oleaceae | *Ligustrum* | *lucidum* | BOP010075 | barcode-057 | CTTATCTTAT |
| Oleaceae | *Ligustrum* | *quihoui* | BOP010314 | barcode-267 | GCCGGGCCGG |
| Oleaceae | *Ligustrum* | *sinense* | BOP010279 | barcode-237 | TCAGCTCAGC |
| Oleaceae | *Ligustrum* | sp. | BOP010277 | barcode-235 | TGGTATGGTA |
| Oleaceae | *Ligustrum* | *vicaryi* | BOP010278 | barcode-236 | ACACGACACG |
| Oleaceae | *Syringa* | *oblata* | BOP010004 | barcode-004 | CTCAGCTCAG |
| Oleaceae | *Syringa* | *oblata* | BOP010005 | barcode-005 | GGGACGGGAC |
| Oleaceae | *Syringa* | *oblata* | BOP010464 | barcode-349 | ATCATATCAT |
| Oleaceae | *Syringa* | *pekinensis* | BOP010002 | barcode-002 | AAGCAAAGCA |
| Oleaceae | *Syringa* | *reticulata subspamurensis* | BOP010153 | barcode-123 | TAGTGTAGTG |
| Oleaceae | *Syringa* | *wolfii* | BOP010003 | barcode-003 | CCAAGCCAAG |
| Pandanaceae | *Pandanus* | *veitchii* | BOP010496 | barcode-375 | ACCCAACCCA |
| Pinaceae | *Pinus* | sp. | BOP010480 | barcode-363 | CCACGCCACG |
| Pinaceae | *Pseudotsuga* | *menziesii* | BOP010481 | barcode-364 | AATACAATAC |
| Platanaceae | *Platanus* | *orientalis* | BOP010462 | barcode-347 | TTAATTTAAT |
| Poaceae | *Fargesia* | sp. | BOP010468 | barcode-352 | GGCTCGGCTC |
| Poaceae | *Indocalamus* | *tessellatus* | BOP010499 | barcode-378 | CTTCGCTTCG |
| Poaceae | *Phyllostachys* | *nigra_var.henonis* | BOP010498 | barcode-377 | TGCAATGCAA |
| Polygonaceae | *Fallopia* | sp. | BOP010146 | barcode-117 | TAATGTAATG |
| Profeaceae | *Grevillea* | *robusta* | BOP010494 | barcode-373 | AAGAAAAGAA |
| Punicaceae | *Punica* | *granatum* | BOP010501 | barcode-380 | CGGGCCGGGC |
| Ranunculaceae | *Clematis* | sp. | BOP010450 | barcode-337 | ACGTCACGTC |
| Rhamnaceae | *Hovenia* | *acerba* | BOP010183 | barcode-152 | GTGGAGTGGA |
| Rhamnaceae | *Rhamnus* | *davurica* | BOP010172 | barcode-141 | GTCGTGTCGT |
| Rhamnaceae | *Rhamnus* | *davurica* | BOP010181 | barcode-150 | TGTGCTGTGC |
| Rhamnaceae | *Rhamnus* | *davurica* | BOP010193 | barcode-162 | GGCCAGGCCA |
| Rhamnaceae | *Rhamnus* | *globosa* | BOP010001 | barcode-001 | TCATATCATA |
| Rhamnaceae | *Rhamnus* | *utilis* | BOP010182 | barcode-151 | CGGGTCGGGT |
| Rhamnaceae | *Sageretia* | *paucicostata* | BOP010189 | barcode-158 | TGCTGTGCTG |
| Rhamnaceae | *Ziziphus* | *jujuba* | BOP010132 | barcode-105 | TCTCGTCTCG |
| Ribesiaceae | *Ribes* | sp. | BOP010190 | barcode-159 | CCTCCCCTCC |
| Ribesiaceae | *Ribes* | sp. | BOP010192 | barcode-161 | CATTGCATTG |
| Rosaceae | *Aria* | sp. | BOP010044 | barcode-028 | TCCAGTCCAG |
| Rosaceae | *Cerasus* | *yedoensis* | BOP010038 | barcode-025 | CCGTACCGTA |
| Rosaceae | *Chaenomeles* | *speciosa* | BOP010159 | barcode-129 | GAAGTGAAGT |
| Rosaceae | *Cotoneaster* | *microphyllus* | BOP010036 | barcode-023 | TATGCTATGC |
| Rosaceae | *Cotoneaster* | sp. | BOP010023 | barcode-018 | TCGATTCGAT |
| Rosaceae | *Cotoneaster* | sp. | BOP010029 | barcode-021 | AGGGGAGGGG |
| Rosaceae | *Cotoneaster* | sp. | BOP010030 | barcode-022 | CGCGTCGCGT |
| Rosaceae | *Cotoneaster* | *submultiflorus* | BOP010185 | barcode-154 | TACCCTACCC |
| Rosaceae | *Crataegus* | *pinnatifida* | BOP010344 | barcode-293 | TATAGTATAG |
| Rosaceae | *Crataegus* | *pinnatifida* | BOP010345 | barcode-294 | TAGGTTAGGT |
| Rosaceae | *Crataegus* | *pinnatifida* | BOP010346 | barcode-295 | GCGAGGCGAG |
| Rosaceae | *Crataegus* | *pinnatifida* | BOP010347 | barcode-296 | CATCACATCA |
| Rosaceae | *Crataegus* | sp. | BOP010018 | barcode-015 | TATACTATAC |
| Rosaceae | *Crataegus* | sp. | BOP010478 | barcode-361 | GCGTTGCGTT |
| Rosaceae | *Cydonia* | *oblanga* | BOP010020 | barcode-017 | ATCCTATCCT |
| Rosaceae | *Cydonia* | sp. | BOP010351 | barcode-299 | TCAGGTCAGG |
| Rosaceae | *Exochorda* | sp. | BOP010472 | barcode-356 | TGAACTGAAC |
| Rosaceae | *Mespilus* | sp. | BOP010017 | barcode-014 | CGCGGCGCGG |
| Rosaceae | *Prinsepia* | *uniflora* | BOP010147 | barcode-118 | TGGAATGGAA |
| Rosaceae | *Prunus* | *davidiana* | BOP010009 | barcode-008 | ATGTCATGTC |
| Rosaceae | *Prunus* | *glandulosa* | BOP010170 | barcode-139 | GTCTTGTCTT |
| Rosaceae | *Prunus* | *glandulosa* | BOP010337 | barcode-287 | CCGTGCCGTG |
| Rosaceae | *Prunus* | *japonica* | BOP010041 | barcode-027 | CGGCACGGCA |
| Rosaceae | *Prunus* | *japonica* | BOP010432 | barcode-319 | ACTCAACTCA |
| Rosaceae | *Prunus* | *padus* | BOP010012 | barcode-010 | GTATGGTATG |
| Rosaceae | *Prunus* | *persica* | BOP010265 | barcode-224 | GAAGGGAAGG |
| Rosaceae | *Prunus* | *persica* | BOP010342 | barcode-292 | CTCTTCTCTT |
| Rosaceae | *Prunus* | *salicina* | BOP010335 | barcode-285 | CTATTCTATT |
| Rosaceae | *Prunus* | *sargentii* | BOP010500 | barcode-379 | ACGGCACGGC |
| Rosaceae | *Prunus* | *serrulata* | BOP010111 | barcode-090 | CGAGACGAGA |
| Rosaceae | *Prunus* | *serrulata* | BOP010333 | barcode-283 | ACGGGACGGG |
| Rosaceae | *Prunus* | *serrulata* | BOP010334 | barcode-284 | GAGAAGAGAA |
| Rosaceae | *Prunus* | *serrulata* | BOP010338 | barcode-288 | AGTAGAGTAG |
| Rosaceae | *Prunus* | *serrulata* | BOP010461 | barcode-346 | CCGAACCGAA |
| Rosaceae | *Prunus* | *serrulata var.lannesiana* | BOP010037 | barcode-024 | GCATTGCATT |
| Rosaceae | *Prunus* | *serrulata var.lannesiana* | BOP010040 | barcode-026 | GCATAGCATA |
| Rosaceae | *Prunus* | *sibirica* | BOP010006 | barcode-006 | TATAATATAA |
| Rosaceae | *Prunus* | *sibirica* | BOP010118 | barcode-094 | AGCGCAGCGC |
| Rosaceae | *Prunus* | sp. | BOP010331 | barcode-282 | GATCAGATCA |
| Rosaceae | *Prunus* | sp. | BOP010336 | barcode-286 | CCGAGCCGAG |
| Rosaceae | *Prunus* | sp. | BOP010339 | barcode-289 | GTTGGGTTGG |
| Rosaceae | *Prunus* | sp. | BOP010341 | barcode-291 | CAACACAACA |
| Rosaceae | *Prunus* | sp. | BOP010477 | barcode-360 | ATGGCATGGC |
| Rosaceae | *Prunus* | sp. | BOP010482 | barcode-365 | CGGTACGGTA |
| Rosaceae | *Prunus* | *subhirtella var.pedula* | BOP010112 | barcode-091 | CTCGACTCGA |
| Rosaceae | *Prunus* | *triloba* | BOP010340 | barcode-290 | GACTGGACTG |
| Rosaceae | *Prunus* | *ussuriensis* | BOP010329 | barcode-280 | GTATCGTATC |
| Rosaceae | *Pyrus* | *betulifolia* | BOP010352 | barcode-300 | AGCCTAGCCT |
| Rosaceae | *Pyrus* | *bretschneideri* | BOP010065 | barcode-047 | TCTTATCTTA |
| Rosaceae | *Pyrus* | *calleryana* | BOP010350 | barcode-298 | TGACTTGACT |
| Rosaceae | *Rosa* | *multiflora* | BOP010453 | barcode-339 | TCTAATCTAA |
| Rosaceae | *Rosa* | sp. | BOP010157 | barcode-127 | GGAATGGAAT |
| Rosaceae | *Rosa* | sp. | BOP010267 | barcode-226 | GATAAGATAA |
| Rosaceae | *Rosa* | *xanthina* | BOP010007 | barcode-007 | CTGGGCTGGG |
| Rosaceae | *Sorbaria* | *sorbifolia* | BOP010264 | barcode-223 | CTGTGCTGTG |
| Rosaceae | *Spiraea* | *chinensis* | BOP010437 | barcode-324 | AAGTTAAGTT |
| Rosaceae | *Spiraea* | *salicifolia* | BOP010066 | barcode-048 | AAGCCAAGCC |
| Rosaceae | *Spiraea* | *salicifolia* | BOP010071 | barcode-053 | AGACCAGACC |
| Rosaceae | *Spiraea* | *salicifolia* | BOP010072 | barcode-054 | TCACATCACA |
| Rosaceae | *Spiraea* | *salicifolia* | BOP010174 | barcode-143 | CTCGGCTCGG |
| Rosaceae | *Spiraea* | *salicifolia* | BOP010438 | barcode-325 | GCTACGCTAC |
| Rosaceae | *Spiraea* | sp. | BOP010360 | barcode-302 | AGTTCAGTTC |
| Rosaceae | *Cydonia* | *oblonga* | BOP010106 | barcode-086 | TTAGGTTAGG |
| Rosaceae | *Spiraea* | sp. | BOP010239 | barcode-203 | AACGAAACGA |
| Rubiaceae | *Leptodermis* | *oblonga* | BOP010280 | barcode-238 | AAGGCAAGGC |
| Rubiaceae | *Paederia* | sp. | BOP010470 | barcode-354 | AACGTAACGT |
| Rutaceae | *Citrus* | *maxima* | BOP010486 | barcode-367 | GCCACGCCAC |
| Rutaceae | *Citrus* | *trifoliata* | BOP010149 | barcode-119 | AGACGAGACG |
| Rutaceae | *Phellodendron* | *chinense* | BOP010196 | barcode-165 | CAATACAATA |
| Rutaceae | *Phellodendron* | sp. | BOP010097 | barcode-078 | TTCTGTTCTG |
| Rutaceae | *Zanthoxylum* | *bungeanum* | BOP010483 | barcode-366 | CAAGCCAAGC |
| Salicaceae | *Populus* | *alba* | BOP010184 | barcode-153 | ATGCTATGCT |
| Salicaceae | *Populus* | *alba var.pyramdalis* | BOP010169 | barcode-138 | AGAATAGAAT |
| Salicaceae | *Populus* | *beijingensis* | BOP010177 | barcode-146 | TCATGTCATG |
| Salicaceae | *Populus* | *canadensis* | BOP010175 | barcode-144 | TCTCATCTCA |
| Salicaceae | *Populus* | *canadensis* | BOP010178 | barcode-147 | GGAGCGGAGC |
| Salicaceae | *Populus* | *candicans* | BOP010187 | barcode-156 | CGCAGCGCAG |
| Salicaceae | *Populus* | *cathayana* | BOP010176 | barcode-145 | TGTTATGTTA |
| Salicaceae | *Populus* | sp. | BOP010024 | barcode-019 | TCCGCTCCGC |
| Salicaceae | *Populus* | sp. | BOP010188 | barcode-157 | GTCTCGTCTC |
| Salicaceae | *Salix* | *babylonica* | BOP010457 | barcode-342 | TTAGATTAGA |
| Salicaceae | *Salix* | *matsudana* | BOP010180 | barcode-149 | TAAGCTAAGC |
| Salicaceae | *Salix* | *matsudana f.pendula* | BOP010137 | barcode-109 | TAGCGTAGCG |
| Salicaceae | *Salix* | sp. | BOP010194 | barcode-163 | TCCCCTCCCC |
| Sapindaceae | *Koelreuteria* | *paniculata* | BOP010051 | barcode-034 | AACTCAACTC |
| Sapindaceae | *Xanthoceras* | *sorbifolia* | BOP010107 | barcode-087 | CACGACACGA |
| Saxifragaceae | *Ribes* | sp. | BOP010049 | barcode-032 | TGCTTTGCTT |
| Schisandraceae | *Schisandra* | *chinensis* | BOP010144 | barcode-116 | TGATCTGATC |
| Scrophulariaceae | *Buddleja* | *lindleyana* | BOP010309 | barcode-263 | CAAAGCAAAG |
| Solanaceae | *Lycium* | *barbarum* | BOP010138 | barcode-110 | ATATCATATC |
| Styracaceae | *Sinojackia* | *xylocarpa* | BOP010105 | barcode-085 | TTGCGTTGCG |
| Styracaceae | *Styrax* | sp. | BOP010242 | barcode-205 | GCACGGCACG |
| Taxaceae | *Amentotaxus* | sp. | BOP010495 | barcode-374 | CAGCACAGCA |
| Tiliaceae | *Tilia* | *amurensis* | BOP010215 | barcode-181 | ACACTACACT |
| Tiliaceae | *Tilia* | *amurensis* | BOP010216 | barcode-182 | ACGCTACGCT |
| Tiliaceae | *Tilia* | *cordata* | BOP010096 | barcode-077 | GCTGTGCTGT |
| Tiliaceae | *Tilia* | *cordata* | BOP010213 | barcode-180 | ACGCAACGCA |
| Tiliaceae | *Tilia* | *tuan* | BOP010095 | barcode-076 | GGGCAGGGCA |
| Tiliaceae | *Tilia* | *tuan* | BOP010208 | barcode-176 | CCCGCCCCGC |
| Tiliaceae | *Tilla* | *phatyphyllos var.cintifolia* | BOP010205 | barcode-173 | TAGGGTAGGG |
| Tiliaceae | *Tilla* | *phatyphyllos var.matabilis* | BOP010206 | barcode-174 | TTACGTTACG |
| Ulmaceae | *Celtis* | *biondii var.heterophylla* | BOP010055 | barcode-038 | TTACCTTACC |
| Ulmaceae | *Celtis* | *bungeana* | BOP010162 | barcode-132 | CCGCTCCGCT |
| Ulmaceae | *Celtis* | *koraiensis* | BOP010303 | barcode-257 | AGGCTAGGCT |
| Ulmaceae | *Hemiptelea* | *davidii* | BOP010300 | barcode-254 | ATAATATAAT |
| Ulmaceae | *Ulmus* | *castaneifolia* | BOP010307 | barcode-261 | AGTTTAGTTT |
| Ulmaceae | *Ulmus* | *laciniata* | BOP010304 | barcode-258 | GGGTAGGGTA |
| Ulmaceae | *Ulmus* | *laevis* | BOP010015 | barcode-013 | GTGCGGTGCG |
| Ulmaceae | *Ulmus* | *lamellosa* | BOP010305 | barcode-259 | AGTATAGTAT |
| Ulmaceae | *Ulmus* | *macrocarpa* | BOP010013 | barcode-011 | ATAAGATAAG |
| Ulmaceae | *Ulmus* | *parvifolia* | BOP010057 | barcode-040 | AGAGTAGAGT |
| Ulmaceae | *Ulmus* | *parvifolia* | BOP010306 | barcode-260 | TTAGTTTAGT |
| Ulmaceae | *Ulmus* | *parvifolia* | BOP010435 | barcode-322 | CCTACCCTAC |
| Ulmaceae | *Ulmus* | *pumila* | BOP010110 | barcode-089 | TCTTGTCTTG |
| Ulmaceae | *Ulmus* | *pumila* | BOP010308 | barcode-262 | TCCGTTCCGT |
| Ulmaceae | *Zelkova* | *schneideriana* | BOP010301 | barcode-255 | CAGGTCAGGT |
| Ulmaceae | *Zelkova* | *serrata* | BOP010302 | barcode-256 | ATGGGATGGG |
| Ulmaceae | *Zelkova* | *sinica* | BOP010447 | barcode-334 | TGTTGTGTTG |
| Verbenaceae | *Callicarpa* | *bodinieri* | BOP010134 | barcode-106 | GCTCGGCTCG |
| Verbenaceae | *Caryopteris* | *clandonensis* | BOP010086 | barcode-068 | ACGACACGAC |
| Verbenaceae | *Clerodendrum* | *trichotomum* | BOP010467 | barcode-351 | CGGATCGGAT |
| Verbenaceae | *Vitex* | *negundo_var.negundo* | BOP010276 | barcode-234 | ACAGGACAGG |
| Vitaceae | *Parthenocissus* | *thomsoni* | BOP010471 | barcode-355 | TAGAATAGAA |
| Vitaceae | *Parthenocissus* | *tricuspidata* | BOP010173 | barcode-142 | AGAAAAGAAA |
| Vitaceae | *Vitis* | sp. | BOP010263 | barcode-222 | AAATCAAATC |
| Vitaceae | *Vitis* | *vinifera* | BOP010143 | barcode-115 | CCACACCACA |
