# Supplementary material for: Method for quick DNA barcode reference library construction
Source: Ecol Evol. 2021 Aug 4;11(17):11627–38. doi: 10.1002/ece3.7788 (PMC8427591; doi:10.1002/ece3.7788)
Supplement: Supplementary file 11 — Table S2 [file ECE3-11-11627-s014.docx]

**Table S2. GenBank accession numbers of reference sequences determined by Sanger sequencing method.**

| **BOP** | ***rbcL*** | ***matK*1** | ***matK*2** | **ITS1** | **ITS2** |
| --- | --- | --- | --- | --- | --- |
| BOP010001 | KP088826 | KP089286 | KP089286 | MT227580 | MT227580 |
| BOP010002 | KP088870 | KP089327 | KP089327 | MT227581 | MT227581 |
| BOP010003 | KP088872 | KP089329 | KP089329 | MT227582 | MT227582 |
| BOP010004 | KP088867 | KP089324 | KP089324 | MT227583 | MT227583 |
| BOP010005 | KP088868 | KP089325 | KP089325 | MT227584 | MT227584 |
| BOP010006 | KP088788 | KP089241 | KP089241 | MT227585 | MT227585 |
| BOP010007 | KP088841 | KP089300 | KP089300 | MT227586 | MT227586 |
| BOP010009 | KP088769 | KP089223 | KP089223 | MT227587 | MT227587 |
| BOP010011 | KP088456 | KP088943 | KP088943 | MT227588 | MT227588 |
| BOP010012 | KP088764 | KP089229 | KP089229 | MT227589 | MT227589 |
| BOP010013 | KP088897 | KP089353 | KP089353 | MT216766 |  |
| BOP010014 | KP088493 | KP088976 | KP088976 | MT216767 |  |
| BOP010015 | KP088895 | KP089351 | KP089351 |  |  |
| BOP010017 | KP088695 | KP089162 | KP089162 |  |  |
| BOP010018 | KP088559 | KP089037 | KP089037 | MT227590 | MT227590 |
| BOP010019 | KP088582 | KP089057 | KP089057 | MT227591 | MT227591 |
| BOP010020 | KP088562 | KP089040 | KP089040 | MT227592 | MT227592 |
| BOP010023 | KP088550 | KP089028 | KP089028 |  |  |
| BOP010024 | KP088755 | KP089215 | KP089215 |  | MT216810 |
| BOP010025 | KP088499 | KP088982 | KP088982 |  |  |
| BOP010029 | KP088551 | KP089029 | KP089029 | MT216768 |  |
| BOP010030 | KP088552 | KP089030 | KP089030 | MT216769 |  |
| BOP010036 | KP088548 | KP089026 | KP089026 |  |  |
| BOP010037 | KP088781 | KP089234 | KP089234 | MT227593 | MT227593 |
| BOP010038 | KP088525 | KP089006 | KP089006 | MT227594 | MT227594 |
| BOP010040 | KP088782 | KP089235 | KP089235 | MT227595 | MT227595 |
| BOP010041 | KP088762 | KP089227 | KP089227 | MT227596 | MT227596 |
| BOP010044 | KP088476 | KP088959 | KP088959 | MT216770 |  |
| BOP010045 | KP088568 | KP089046 | KP089046 | MT227597 | MT227597 |
| BOP010046 | KP088710 | KP089172 | KP089172 | MT227598 | MT227598 |
| BOP010048 | KP088676 | KP089145 | KP089145 |  | MT216811 |
| BOP010049 | KP088834 | KP089294 | KP089294 |  | MT216812 |
| BOP010050 | KP088472 | KP088955 | KP088955 | MT227599 |  |
| BOP010051 | KP088643 | KP089116 | KP089116 |  |  |
| BOP010052 | KP088600 | KP089074 | KP089074 | MT227600 |  |
| BOP010053 | KP088665 | KP089137 | KP089137 |  |  |
| BOP010054 | KP088622 | KP089096 | KP089096 | MT216771 |  |
| BOP010055 | KP088520 | KP089002 | KP089002 | MT227601 | MT227601 |
| BOP010056 | KP088602 | KP089076 | KP089076 | MT227602 | MT227602 |

Data Accessibility table Continued

| **BOP** | ***rbcL*** | ***matK*1** | ***matK*2** | **ITS1** | **ITS2** |
| --- | --- | --- | --- | --- | --- |
| BOP010057 | KP088898 | KP088121 | KP088121 |  |  |
| BOP010059 | KP088528 | KP088123 | KP088123 | MT227603 | MT227603 |
| BOP010060 | KP088526 | KP088124 | KP088124 | MT227604 | MT227604 |
| BOP010061 | KP088633 | KP089107 | KP089107 | MT227605 | MT227605 |
| BOP010062 | KP088853 | KP089311 | KP089311 | MT227606 | MT227606 |
| BOP010063 | KP088507 | KP088990 | KP088990 | MT227607 | MT227607 |
| BOP010064 | KP088547 | KP089025 | KP089025 | MT227608 | MT227608 |
| BOP010065 | KP088778 | KP089258 | KP089258 |  |  |
| BOP010066 | KP088860 | KP089318 | KP089318 |  |  |
| BOP010067 | KP088481 | KP088964 | KP088964 | MT227609 | MT227609 |
| BOP010068 | KP088491 | KP088974 | KP088974 | MT227610 | MT227610 |
| BOP010069 | KP088480 | KP088963 | KP088963 | MT227611 | MT227611 |
| BOP010070 | KP088486 | KP088969 | KP088969 | MT227612 | MT227612 |
| BOP010071 | KP088861 | KP089319 | KP089319 |  | MT216813 |
| BOP010072 | KP088862 | KP089320 | KP089320 |  |  |
| BOP010073 | KP088484 | KP088967 | KP088967 | MT227613 | MT227613 |
| BOP010074 | KP088832 | KP089292 | KP089292 | MT227614 | MT227614 |
| BOP010075 | KP088653 | KP089126 | KP089126 | MT227615 | MT227615 |
| BOP010076 | KP088487 | KP088970 | KP088970 | MT227616 | MT227616 |
| BOP010077 | KP088482 | KP088965 | KP088965 | MT227617 | MT227617 |
| BOP010078 | KP088478 | KP088961 | KP088961 | MT227618 | MT227618 |
| BOP010079 | KP088490 | KP088973 | KP088973 | MT227619 | MT227619 |
| BOP010080 | KP088489 | KP088972 | KP088972 | MT227620 | MT227620 |
| BOP010081 | KP088483 | KP088966 | KP088966 | MT227621 | MT227621 |
| BOP010082 | KP088479 | KP088962 | KP088962 | MT227622 | MT227622 |
| BOP010083 | KP088485 | KP088968 | KP088968 | MT227623 | MT227623 |
| BOP010084 | KP088488 | KP088971 | KP088971 | MT227624 | MT227624 |
| BOP010085 | KP088614 | KP089088 | KP089088 | MT227625 | MT227625 |
| BOP010086 | KP088512 | KP088995 | KP088995 |  |  |
| BOP010087 | KP088590 | KP089064 | KP089064 |  | MT216814 |
| BOP010088 | KP088854 | KP089312 | KP089312 |  | MT216815 |
| BOP010090 | KP088630 | KP089104 | KP089104 | MT227626 | MT227626 |
| BOP010091 | KP088448 | KP088935 | KP088935 | MT227627 | MT227627 |
| BOP010092 | KP088681 | KP089149 | KP089149 |  |  |
| BOP010093 | KP088541 | KP089019 | KP089019 |  |  |
| BOP010094 | KP088604 | KP089078 | KP089078 | MT227628 | MT227628 |
| BOP010095 | KP088886 | KP089343 | KP089343 |  |  |
| BOP010096 | KP088884 | KP089341 | KP089341 | MT227629 | MT227629 |
| BOP010097 | KP088707 | KP089169 | KP089169 | MT216772 |  |
| BOP010098 | KP088677 | KP089146 | KP089146 | MT216773 |  |

Data Accessibility table Continued

| **BOP** | ***rbcL*** | ***matK*1** | ***matK*2** | **ITS1** | **ITS2** |
| --- | --- | --- | --- | --- | --- |
| BOP010099 | KP088659 | KP089132 | KP089132 |  |  |
| BOP010100 | KP088682 | KP089150 | KP089150 | MT216774 |  |
| BOP010101 | KP088477 | KP088960 | KP088960 |  |  |
| BOP010102 | KP088922 | KP089374 | KP089374 | MT227630 | MT227630 |
| BOP010104 | KP088679 | KP089148 | KP089148 | MT227631 | MT227631 |
| BOP010105 | KP088852 | KP088151 | KP088151 |  |  |
| BOP010106 | |  |  |  |  |
| BOP010107 | KP088923 | KP089375 | KP089375 | MT227632 | MT227632 |
| BOP010108 | KP088505 | KP088988 | KP088988 | MT216775 |  |
| BOP010110 | KP088901 | KP089355 | KP089355 |  | MT216816 |
| BOP010111 | KP088783 | KP089236 | KP089236 | MT227633 | MT227633 |
| BOP010112 | KP088774 | KP089249 | KP089249 | MT227634 | MT227634 |
| BOP010116 | KP088635 | KP089108 | KP089108 | MT227635 | MT227635 |
| BOP010117 | KP088636 | KP089109 | KP089109 | MT227636 | MT227636 |
| BOP010118 | KP088789 | KP089242 | KP089242 | MT227637 | MT227637 |
| BOP010119 | KP088578 | KP089053 | KP089053 | MT227638 | MT227638 |
| BOP010120 | KP088613 | KP089087 | KP089087 | MT227639 | MT227639 |
| BOP010121 | KP088601 | KP089075 | KP089075 |  | MT216817 |
| BOP010122 | KP088661 | KP089134 | KP089134 |  |  |
| BOP010125 | KP088924 | KP089376 | KP089376 |  |  |
| BOP010126 | KP088588 | KP089062 | KP089062 | MT216776 | MT216818 |
| BOP010127 | KP088591 | KP089065 | KP089065 | MT227640 | MT227640 |
| BOP010128 | KP088592 | KP089066 | KP089066 | MT227641 | MT227641 |
| BOP010129 | KP088589 | KP089063 | KP089063 | MT216777 |  |
| BOP010131 | KP088586 | KP089060 | KP089060 |  |  |
| BOP010132 | KP088929 | KP089379 | KP089379 | MT227642 | MT227642 |
| BOP010134 | KP088502 | KP088987 | KP088987 | MT227643 | MT227643 |
| BOP010135 | KP088504 | KP088985 | KP088985 |  |  |
| BOP010136 | KP088492 | KP088975 | KP088975 | MT227644 | MT227644 |
| BOP010137 | KP088846 | KP089305 | KP089305 |  |  |
| BOP010138 | KP088673 | KP089142 | KP089142 | MT216778 |  |
| BOP010139 | |  |  |  |  |
| BOP010140 | KP088461 | KP088946 | KP088946 | MT227645 | MT227645 |
| BOP010141 | KP088459 | MT157359 | MT157367 | MT227646 | MT227646 |
| BOP010142 | KP088458 | MT157360 | MT157360 | MT227647 | MT227647 |
| BOP010143 | KP088918 | KP089371 | KP089371 |  |  |
| BOP010144 | KP088851 | KP089310 | KP089310 |  |  |
| BOP010146 | |  |  |  |  |
| BOP010147 | KP088761 | KP089221 | KP089221 | MT227648 | MT227648 |
| BOP010149 | KP088538 | KP089016 | KP089016 |  |  |

Data Accessibility table Continued

| **BOP** | ***rbcL*** | ***matK*1** | ***matK*2** | **ITS1** | **ITS2** |
| --- | --- | --- | --- | --- | --- |
| BOP010150 | KP088569 | KP089047 | KP089047 |  |  |
| BOP010151 | KP088570 | KP089048 | KP089048 |  |  |
| BOP010152 | KP088663 | KP089135 | KP089135 |  |  |
| BOP010153 | KP088871 | KP089328 | KP089328 | MT227649 | MT227649 |
| BOP010154 | KP088658 | KP089131 | KP089131 |  |  |
| BOP010155 | KP088463 | KP088948 | KP088948 |  |  |
| BOP010156 | KP088678 | KP089147 | KP089147 | MT216779 |  |
| BOP010157 | KP088840 | KP089299 | KP089299 | MT227650 | MT227650 |
| BOP010158 | KP088906 | KP089360 | KP089360 |  | MT216819 |
| BOP010159 | KP088530 | KP089009 | KP089009 |  |  |
| BOP010160 | KP088829 | KP089289 | KP089289 | MT227651 | MT227651 |
| BOP010161 | KP088799 | MT157364 | MT157374 | MT227652 | MT227652 |
| BOP010162 | KP088522 | KP089004 | KP089004 | MT227653 | MT227653 |
| BOP010163 | KP088831 | KP089291 | KP089291 | MT227654 | MT227654 |
| BOP010164 | KP088565 | KP089043 | KP089043 |  |  |
| BOP010166 | KP088907 | KP089361 | KP089361 | MT227655 | MT227655 |
| BOP010167 | KP088675 | KP089144 | KP089144 | MT227656 | MT227656 |
| BOP010168 | |  |  |  |  |
| BOP010169 | KP088751 | KP089211 | KP089211 |  |  |
| BOP010170 | KP088771 | KP089225 | KP089225 | MT227657 | MT227657 |
| BOP010171 | KP088646 | KP089119 | KP089119 | MT227658 | MT227658 |
| BOP010172 | KP088823 | KP089283 | KP089283 | MT227659 | MT227659 |
| BOP010173 | KP088704 |  |  |  |  |
| BOP010174 | KP088863 | KP089321 | KP089321 |  |  |
| BOP010175 | KP088758 | KP089218 | KP089218 |  |  |
| BOP010176 | KP088754 | KP089214 | KP089214 |  |  |
| BOP010177 | KP088757 | KP089217 | KP089217 |  |  |
| BOP010178 | KP088759 | KP089219 | KP089219 |  |  |
| BOP010179 | KP088583 | KP089058 | KP089058 | MT227660 | MT227660 |
| BOP010180 | KP088847 | KP089306 | KP089306 |  | MT216820 |
| BOP010181 | KP088824 | KP089284 | KP089284 | MT227661 | MT227661 |
| BOP010182 | KP088827 | KP089287 | KP089287 | MT227662 | MT227662 |
| BOP010183 | KP088631 | KP089105 | KP089105 | MT227663 | MT227663 |
| BOP010184 | KP088752 | KP089212 | KP089212 | MT216780 |  |
| BOP010185 | KP088553 | KP089031 | KP089031 | MT216781 |  |
| BOP010186 | KP088573 | KP088215 | KP088215 |  |  |
| BOP010187 | KP088753 | KP089213 | KP089213 |  |  |
| BOP010188 | KP088756 | KP089216 | KP089216 |  |  |
| BOP010189 | KP088844 | KP089303 | KP089303 | MT227664 | MT227664 |
| BOP010190 | KP088835 | KP089295 | KP089295 | MT227665 | MT227665 |

Data Accessibility table Continued

| **BOP** | ***rbcL*** | ***matK*1** | ***matK*2** | **ITS1** | **ITS2** |
| --- | --- | --- | --- | --- | --- |
| BOP010191 | KP088674 | KP089143 | KP089143 | MT227666 | MT227666 |
| BOP010192 | KP088836 | KP089296 | KP089296 | MT227667 | MT227667 |
| BOP010193 | KP088825 | KP089285 | KP089285 | MT227668 | MT227668 |
| BOP010194 | KP088848 | KP089307 | KP089307 | MT227669 | MT227669 |
| BOP010195 | KP088457 | KP088944 | KP088944 | MT227670 | MT227670 |
| BOP010196 | KP088706 | KP089168 | KP089168 |  |  |
| BOP010198 | KP088453 | KP088940 | KP088940 |  | MT216821 |
| BOP010199 | KP088449 | KP088936 | KP088936 | MT227671 | MT227671 |
| BOP010200 | KP088455 | KP088942 | KP088942 | MT227672 | MT227672 |
| BOP010201 | KP088452 | KP088939 | KP088939 | MT227673 | MT227673 |
| BOP010202 | KP088891 | KP089347 | KP089347 | MT227674 | MT227674 |
| BOP010203 | KP088833 | KP089293 | KP089293 | MT227675 | MT227675 |
| BOP010204 | KP088746 | KP089206 | KP089206 | MT227676 | MT227676 |
| BOP010205 | KP088888 | KP089345 | KP089345 | MT227677 | MT227677 |
| BOP010206 | KP088889 | KP089346 | KP089346 | MT227678 | MT227678 |
| BOP010207 | |  |  |  |  |
| BOP010208 | KP088887 | KP089344 | KP089344 |  |  |
| BOP010209 | KP088451 | KP088938 | KP088938 |  |  |
| BOP010210 | KP088450 | KP088937 | KP088937 | MT227679 | MT227679 |
| BOP010211 | KP088454 | KP088941 | KP088941 | MT227680 | MT227680 |
| BOP010213 | KP088885 | KP089342 | KP089342 | MT227681 | MT227681 |
| BOP010215 | KP088881 | KP089338 | KP089338 |  |  |
| BOP010216 | KP088882 | KP089339 | KP089339 |  |  |
| BOP010218 | KP088709 | KP089171 | KP089171 |  | MT216822 |
| BOP010219 | KP088708 | KP089170 | KP089170 |  |  |
| BOP010220 | KP088711 | KP089173 | KP089173 |  |  |
| BOP010221 | KP088712 | KP089174 | KP089174 |  |  |
| BOP010222 | KP088713 | KP089175 | KP089175 |  |  |
| BOP010223 | KP088500 | KP088983 | KP088983 |  |  |
| BOP010224 | KP088609 | KP089083 | KP089083 | MT216782 |  |
| BOP010226 | KP088620 | KP089094 | KP089094 |  |  |
| BOP010227 | KP088518 | KP088999 | KP088999 | MT227682 | MT227682 |
| BOP010228 | KP088619 | KP089093 | KP089093 | MT227683 | MT227683 |
| BOP010229 | KP088610 | KP089084 | KP089084 | MT227684 | MT227684 |
| BOP010230 | KP088920 | MT157368 | MT157368 | MT227685 | MT227685 |
| BOP010231 | KP088536 | KP089015 | KP089015 | MT227686 | MT227686 |
| BOP010232 | KP088617 | KP089091 | KP089091 | MT227687 | MT227687 |
| BOP010233 | KP088616 | KP089090 | KP089090 | MT227688 | MT227688 |
| BOP010234 | KP088618 | KP089092 | KP089092 | MT227689 | MT227689 |
| BOP010235 | KP088611 | KP089085 | KP089085 | MT216783 | MT216823 |

Data Accessibility table Continued

| **BOP** | ***rbcL*** | ***matK*1** | ***matK*2** | **ITS1** | **ITS2** |
| --- | --- | --- | --- | --- | --- |
| BOP010236 | KP088612 | KP089086 | KP089086 | MT227690 | MT227690 |
| BOP010237 | KP088608 | KP089082 | KP089082 | MT227691 | MT227691 |
| BOP010238 | KP088516 | KP088997 | KP088997 | MT227692 | MT227692 |
| BOP010239 | |  |  |  |  |
| BOP010241 | KP088615 | KP089089 | KP089089 | MT227693 | MT227693 |
| BOP010242 | KP088866 | KP088261 | KP088261 | MT227694 | MT227694 |
| BOP010243 | KP088574 | KP088262 | KP088262 |  |  |
| BOP010244 | KP088571 | KP088263 | KP088263 |  |  |
| BOP010245 | KP088910 | KP089364 | KP089364 | MT216784 | MT216824 |
| BOP010246 | KP088914 | KP089368 | KP089368 | MT227695 | MT227695 |
| BOP010247 | KP088915 | KP089369 | KP089369 | MT227696 | MT227696 |
| BOP010248 | KP088921 | KP089373 | KP089373 | MT227697 | MT227697 |
| BOP010249 | KP088464 | KP088949 | KP088949 | MT227698 | MT227698 |
| BOP010250 | KP088462 | KP088947 | KP088947 | MT227699 | MT227699 |
| BOP010252 | KP088587 | KP089061 | KP089061 |  |  |
| BOP010254 | KP088911 | KP089365 | KP089365 | MT227700 | MT227700 |
| BOP010255 | KP088913 | KP089367 | KP089367 | MT216785 | MT216825 |
| BOP010257 | KP088904 | KP089358 | KP089358 | MT216786 | MT216826 |
| BOP010258 | KP088912 | KP089366 | KP089366 |  | MT216827 |
| BOP010259 | KP088905 | KP089359 | KP089359 |  | MT216828 |
| BOP010260 | KP088908 | KP089362 | KP089362 |  | MT216829 |
| BOP010262 | KP088903 | KP089357 | KP089357 |  | MT216830 |
| BOP010263 | KP088917 | KP089370 | KP089370 | MT227701 | MT227701 |
| BOP010264 | KP088856 | KP089314 | KP089314 | MT227702 | MT227702 |
| BOP010265 | KP088765 | KP089230 | KP089230 | MT216787 | MT216831 |
| BOP010266 | KP088506 | KP088989 | KP088989 |  |  |
| BOP010267 | KP088839 | KP088283 | KP088283 | MT227703 | MT227703 |
| BOP010269 | KP088849 | KP089308 | KP089308 |  |  |
| BOP010270 | KP088540 | KP089018 | KP089018 | MT227704 | MT227704 |
| BOP010271 | KP088807 | KP089266 | KP089266 | MT227705 | MT227705 |
| BOP010272 | KP088855 | KP089313 | KP089313 | MT227706 | MT227706 |
| BOP010273 | KP088606 | KP089080 | KP089080 | MT227707 | MT227707 |
| BOP010274 | KP088605 | KP089079 | KP089079 | MT227708 | MT227708 |
| BOP010275 | KP088603 | KP089077 | KP089077 | MT227709 | MT227709 |
| BOP010276 | KP088916 | KP088291 | KP088291 | MT227710 | MT227710 |
| BOP010277 | KP088656 | KP089129 | KP089129 | MT227711 | MT227711 |
| BOP010278 | KP088657 | KP089130 | KP089130 | MT227712 | MT227712 |
| BOP010279 | KP088655 | KP089128 | KP089128 | MT227713 | MT227713 |
| BOP010280 | KP088652 | KP089125 | KP089125 | MT227714 | MT227714 |
| BOP010281 | KP088668 | MT157361 | MT157369 |  | MT216832 |

Data Accessibility table Continued

| **BOP** | ***rbcL*** | ***matK*1** | ***matK*2** | **ITS1** | **ITS2** |
| --- | --- | --- | --- | --- | --- |
| BOP010282 | KP088497 | KP088980 | KP088980 | MT227715 | MT227715 |
| BOP010283 | KP088669 | KP089138 | KP089138 | MT216788 | MT216833 |
| BOP010286 | |  |  |  |  |
| BOP010287 | KP088662 | MT157372 | MT157372 |  | MT216834 |
| BOP010288 | KP088667 | MT157362 | MT157370 | MT227716 | MT227716 |
| BOP010290 | KP088627 | KP089101 | KP089101 | MT227717 | MT227717 |
| BOP010291 | KP088666 | MT157371 | MT157371 |  | MT216835 |
| BOP010292 | KP088670 | KP089139 | KP089139 | MT227718 | MT227718 |
| BOP010293 | KP088501 | KP088984 | KP088984 |  |  |
| BOP010294 | KP088672 | KP089141 | KP089141 |  |  |
| BOP010295 | KP088503 | KP088986 | KP088986 |  | MT216836 |
| BOP010296 | KP088798 | KP089255 | KP089255 | MT227719 | MT227719 |
| BOP010297 | KP088797 | KP089254 | KP089254 | MT227720 | MT227720 |
| BOP010299 | KP088510 | KP088993 | KP088993 | MT227721 | MT227721 |
| BOP010300 | KP088629 | KP089103 | KP089103 |  | MT216837 |
| BOP010301 | KP088926 | KP088304 | KP088304 | MT227722 | MT227722 |
| BOP010302 | KP088927 | KP089378 | KP089378 | MT216789 | MT216838 |
| BOP010303 | KP088523 | KP089005 | KP089005 | MT227723 | MT227723 |
| BOP010304 | KP088894 | KP088307 | KP088307 |  |  |
| BOP010305 | KP088896 | KP089352 | KP089352 | MT227724 | MT227724 |
| BOP010306 | KP088899 | KP089354 | KP089354 |  | MT216839 |
| BOP010307 | KP088893 | KP089350 | KP089350 |  | MT216840 |
| BOP010308 | KP088902 | KP089356 | KP089356 | MT227725 | MT227725 |
| BOP010309 | KP088498 | KP088981 | KP088981 | MT216790 | MT216841 |
| BOP010311 | KP088469 | KP088953 | KP088953 | MT227726 | MT227726 |
| BOP010312 | KP088543 | KP089021 | KP089021 | MT227727 | MT227727 |
| BOP010313 | KP088544 | KP089022 | KP089022 |  |  |
| BOP010314 | KP088654 | KP089127 | KP089127 | MT227728 | MT227728 |
| BOP010315 | KP088566 | KP089044 | KP089044 |  | MT216842 |
| BOP010316 | KP088567 | KP089045 | KP089045 | MT227729 | MT227729 |
| BOP010317 | KP088496 | KP088979 | KP088979 | MT227730 | MT227730 |
| BOP010318 | KP088697 | KP089164 | KP089164 | MT227731 | MT227731 |
| BOP010319 | KP088579 | KP089054 | KP089054 | MT227732 | MT227732 |
| BOP010320 | KP088580 | KP089055 | KP089055 | MT227733 | MT227733 |
| BOP010322 | KP088475 | KP088958 | KP088958 | MT227734 | MT227734 |
| BOP010323 | KP088581 | KP089056 | KP089056 | MT227735 | MT227735 |
| BOP010324 | KP088470 | KP088954 | KP088954 | MT227736 | MT227736 |
| BOP010325 | KP088641 | KP089114 | KP089114 | MT227737 | MT227737 |
| BOP010326 | KP088577 | KP089052 | KP089052 | MT227738 | MT227738 |
| BOP010328 | KP088513 | KP088328 | KP088328 |  |  |

Data Accessibility table Continued

| **BOP** | ***rbcL*** | ***matK*1** | ***matK*2** | **ITS1** | **ITS2** |
| --- | --- | --- | --- | --- | --- |
| BOP010329 | KP088776 | KP089252 | KP089252 |  |  |
| BOP010330 | KP088546 | KP089024 | KP089024 | MT227739 | MT227739 |
| BOP010331 | KP088794 | KP089248 | KP089248 | MT227740 | MT227740 |
| BOP010333 | KP088784 | KP089237 | KP089237 | MT227741 | MT227741 |
| BOP010334 | KP088785 | KP089238 | KP089238 | MT227742 | MT227742 |
| BOP010335 | KP088767 | KP089232 | KP089232 | MT227743 | MT227743 |
| BOP010336 | KP088792 | KP089246 | KP089246 | MT227744 | MT227744 |
| BOP010337 | KP088772 | KP089226 | KP089226 | MT227745 | MT227745 |
| BOP010338 | KP088786 | KP089239 | KP089239 | MT227746 | MT227746 |
| BOP010339 | KP088793 | KP089247 | KP089247 | MT227747 | MT227747 |
| BOP010340 | KP088795 | KP089251 | KP089251 | MT227748 | MT227748 |
| BOP010341 | KP088791 | KP089245 | KP089245 | MT227749 | MT227749 |
| BOP010342 | KP088766 | KP089231 | KP089231 | MT216791 | MT216843 |
| BOP010344 | KP088555 | KP089033 | KP089033 |  |  |
| BOP010345 | KP088556 | KP089034 | KP089034 | MT216792 |  |
| BOP010346 | KP088557 | KP089035 | KP089035 | MT216793 |  |
| BOP010347 | KP088558 | KP089036 | KP089036 | MT216794 |  |
| BOP010348 | KP088637 | KP089110 | KP089110 | MT227750 | MT227750 |
| BOP010350 | KP088779 | KP089259 | KP089259 | MT216795 |  |
| BOP010351 | KP088563 | KP089041 | KP089041 | MT216796 |  |
| BOP010352 | KP088777 | KP089257 | KP089257 | MT216797 |  |
| BOP010359 | KP088830 | KP089290 | KP089290 | MT227751 | MT227751 |
| BOP010360 | KP088865 | KP089323 | KP089323 | MT216798 |  |
| BOP010416 | KP088444 | KP088930 | KP088930 |  |  |
| BOP010417 | KP088818 | KP089278 | KP089278 | MT216799 | MT216844 |
| BOP010418 | KP088814 | KP089274 | KP089274 | MT216800 | MT216845 |
| BOP010419 | KP088815 | KP089275 | KP089275 | MT216801 | MT216846 |
| BOP010420 | KP088808 | KP089267 | KP089267 | MT227752 | MT227752 |
| BOP010421 | KP088801 | KP089260 | KP089260 | MT227753 | MT227753 |
| BOP010422 | KP088802 | KP089261 | KP089261 | MT227754 | MT227754 |
| BOP010423 | KP088804 | KP089263 | KP089263 | MT227755 | MT227755 |
| BOP010424 | KP088821 | KP089281 | KP089281 | MT216802 | MT216847 |
| BOP010425 | KP088819 | KP089279 | KP089279 | MT227756 | MT227756 |
| BOP010426 | KP088809 | KP089268 | KP089268 | MT227757 | MT227757 |
| BOP010427 | KP088811 | KP089270 | KP089270 | MT227758 | MT227758 |
| BOP010428 | KP088812 | KP089272 | KP089272 | MT227759 | MT227759 |
| BOP010429 | KP088813 | KP089273 | KP089273 |  | MT216848 |
| BOP010430 | KP088820 | KP089280 | KP089280 |  | MT216849 |
| BOP010431 | KP088810 | KP089269 | KP089269 |  | MT216850 |
| BOP010432 | KP088763 | KP089228 | KP089228 | MT227760 | MT227760 |

Data Accessibility table Continued

| **BOP** | ***rbcL*** | ***matK*1** | ***matK*2** | **ITS1** | **ITS2** |
| --- | --- | --- | --- | --- | --- |
| BOP010433 | KP088816 | KP089276 | KP089276 | MT227761 | MT227761 |
| BOP010434 | KP088509 | KP088992 | KP088992 | MT227762 | MT227762 |
| BOP010435 | KP088900 | KP088378 | KP088378 | MT227763 | MT227763 |
| BOP010436 | KP088508 | KP088991 | KP088991 | MT227764 | MT227764 |
| BOP010437 | KP088857 | KP089315 | KP089315 |  |  |
| BOP010438 | KP088864 | KP089322 | KP089322 | MT227765 | MT227765 |
| BOP010439 | KP088805 | KP089264 | KP089264 | MT216803 | MT216851 |
| BOP010440 | KP088817 | KP089277 | KP089277 | MT227766 | MT227766 |
| BOP010441 | KP088806 | KP089265 | KP089265 | MT227767 | MT227767 |
| BOP010442 | KP088803 | KP089262 | KP089262 | MT216804 | MT216852 |
| BOP010443 | KP089271 |  |  |  | MT216853 |
| BOP010444 | KP088623 | KP089097 | KP089097 |  |  |
| BOP010445 | KP088822 | KP089282 | KP089282 | MT227768 | MT227768 |
| BOP010446 | KP088545 | KP089023 | KP089023 |  |  |
| BOP010447 | KP088928 | KP088387 | KP088387 |  |  |
| BOP010448 | KP088671 | KP089140 | KP089140 |  |  |
| BOP010449 | KP088664 | KP089136 | KP089136 |  |  |
| BOP010450 | |  |  |  |  |
| BOP010452 | KP088632 | KP089106 | KP089106 | MT227769 | MT227769 |
| BOP010453 | KP088837 | KP089297 | KP089297 | MT227770 | MT227770 |
| BOP010454 | KP088879 | KP089336 | KP089336 | MT227771 | MT227771 |
| BOP010455 | KP088680 | KP088391 | KP088391 |  |  |
| BOP010457 | KP088845 | KP089304 | KP089304 | MT227772 | MT227772 |
| BOP010458 | KP088517 | KP088998 | KP088998 | MT227773 | MT227773 |
| BOP010459 | KP088527 | KP089007 | KP089007 | MT227774 | MT227774 |
| BOP010460 | KP088694 | KP089161 | KP089161 |  | MT216854 |
| BOP010461 | KP088787 | KP089240 | KP089240 | MT227775 | MT227775 |
| BOP010462 | KP088748 | KP089208 | KP089208 | MT227776 | MT227776 |
| BOP010463 | KP088701 | KP089166 | KP089166 |  |  |
| BOP010464 | KP088869 | KP089326 | KP089326 | MT227777 | MT227777 |
| BOP010465 | KP088714 | KP089176 | KP089176 | MT227778 | MT227778 |
| BOP010467 | KP088539 | KP089017 | KP089017 | MT227779 | MT227779 |
| BOP010468 | KP088597 | KP089071 | KP089071 | MT216805 |  |
| BOP010469 | |  |  |  |  |
| BOP010470 | |  |  |  |  |
| BOP010471 | KP088703 | KP089167 | KP089167 | MT227780 | MT227780 |
| BOP010472 | KP088596 | KP089070 | KP089070 | MT227781 | MT227781 |
| BOP010473 | KP088890 | MT157363 | MT157375 | MT227782 | MT227782 |
| BOP010474 | KP088598 | KP089072 | KP089072 | MT227783 | MT227783 |
| BOP010476 | KP088626 | KP089100 | KP089100 |  | MT216855 |

Data Accessibility table Continued

| **BOP** | ***rbcL*** | ***matK*1** | ***matK*2** | **ITS1** | **ITS2** |
| --- | --- | --- | --- | --- | --- |
| BOP010477 | KP088780 | KP089243 | KP089243 | MT216806 |  |
| BOP010478 | KP088560 | KP089038 | KP089038 |  |  |
| BOP010479 | KP088460 | KP088945 | KP088945 | MT227784 | MT227784 |
| BOP010480 | KP088739 | KP089199 | KP089199 |  |  |
| BOP010481 | KP088796 | KP089253 | KP089253 |  |  |
| BOP010482 | KP088790 | KP089244 | KP089244 |  | MT216856 |
| BOP010483 | KP088925 | KP089377 | KP089377 | MT227785 | MT227785 |
| BOP010486 | KP088537 | KP088410 | KP088410 | MT227786 | MT227786 |
| BOP010488 | KP088873 | KP089330 | KP089330 | MT227787 | MT227787 |
| BOP010490 | |  |  |  |  |
| BOP010491 | KP088699 | KP088413 | KP088413 |  |  |
| BOP010492 | KP088514 | MT157373 | MT157373 | MT216807 |  |
| BOP010493 | KP088599 | KP089073 | KP089073 | MT227788 | MT227788 |
| BOP010494 | KP088624 | KP089098 | KP089098 |  |  |
| BOP010495 | KP088471 | KP088934 | KP088934 |  |  |
| BOP010496 | KP088702 | KP088416 | KP088416 | MT227789 | MT227789 |
| BOP010497 | KP088651 | KP089124 | KP089124 |  |  |
| BOP010498 | KP088716 | MT157365 | MT157376 | MT216808 |  |
| BOP010499 | KP088634 | MT157366 | MT157377 |  |  |
| BOP010500 | KP088773 | KP089233 | KP089233 |  | MT216857 |
| BOP010501 | KP088800 | KP089256 | KP089256 | MT227790 | MT227790 |
