# Supplementary material for: Method for quick DNA barcode reference library construction
Source: Ecol Evol. 2021 Aug 4;11(17):11627–38. doi: 10.1002/ece3.7788 (PMC8427591; doi:10.1002/ece3.7788)
Supplement: Supplementary file 12 — Supporting Document S1 [file ECE3-11-11627-s018.docx]

**Cotu Master User’s Manual**

**Yanlei Liu**

**Cotu method**

In order to support accurate molecular identification of organisms by means of DNA barcoding, a reference library with high species coverage needs to be constructed as quick and cheap as possible. To reach this goal, high throughput sequencing platforms are indispensable to speed up the processes and lower the costs. Illumina Hiseq PE250 is currently the right platform for conventional DNA barcodes.

The software currently available has two imperfections. One is the potential of a wrong read chosen as a representative of a sequence, and the other is that the representative is shorter than expected. In order to improve the accuracy and length of output sequences, we developed a new NGS data processing method called “Cotu” under the majority consensus rule (the letter “C” stands for consensus, and “otu” follows the “Zotu” fashion). The major features of the Cotu method are (1) consensus sequences of all possibly correct reads instead of representatives; and (2) elimination of insertions from occasional reads under majority rule and sequence length extensions at both ends of alignments. The qualities of the beginning and ending bases are usually low and frequently trimmed off, which causes uneven alignments. If the majority rule was used for this situation, shortened consensus sequences would likely be created. We, therefore, apply a threshold of bases on a site (e.g. 20%) at both ends. In order to reduce gaps in an alignment, fake bases with less than 10% of total bases in a position are automatically removed before the application of majority rule. The program calculates the number of bases in three consecutive base positions and sets a threshold. If a site in the beginning and ending regions has bases less than the threshold, the site is considered an incorrect insertion and it is deleted; and if a site has bases more than the threshold, the site is considered normal and the majority rule is applied. For bases between both ends, if bases are fewer than 50%, the site will be considered as a real gap, otherwise, the gaps will be ignored and the position will be treated as normal sequence with the applications of majority rule of bases (ignoring gaps).

**To Cotu users**

This package is just suitable for 64 bite windows operating system.

Please ensure that python3.7 or upper version was installed.

Please have mafft, usearch and vsearch installed in your computer

Citations to other programs (mafft, usearch and vsearch) are recommended besides Cotu.

Please read readme.txt in the Cotu Master folder before going ahead.

**How to use Cotu-generator.py**

Open cmd.exe, change to Cotu Master working folder and input:

python Cotu-generator.py –help

You will see information below:

useage: python Cotu-generator.py –i input_file [options] For example:

python Cotu-generator.py -i alignment-file.fasta –o output-filename –t Single -d 5 -id1 0.2 -id2 0.5

optional arguments:

-h, --help show this help message and exit

-i: Input file.

-o Output sample name

-t Type of copy [Single or Multi]

-cp The ranking of copy number

-d: The minimum sequencing depth, sample with sequencing depth lower than 5 will be dropt.

-id1: The first sequencing depth strategy, in a certain region of two ends, base number counted larger than the parameter given will ignore gaps and conducted the majority for consensus generation.

-id2: The second sequencing depth strategy, in a certain region of the middle, base number counted larger than the parameter given will ignore gaps and conducted the majority for consensus generation. If not, a gap will be produced in the certain position.

Practical examples:

Three test data in fastq formation were provided.

Open the results folder and find BOP010001.fasta and run the command below:

python Cotu-generator.py -i BOP010001.fasta –o BOP010001 –t Single -d 10 -id1 0.2 -id2 0.5

###In the beginning and ending region self-learned by Cotu-generator, 0.2 parameter will be used while in the middle region 0.5 parameter will be used for Cotu generation.

For users who want to generate single Cotu for all fasta files in a folder, we provide a cycle command: Please copy Cotu-generator.py into the folder before you run the command.

for %i in (*.fasta); do python Cotu-generator.py -i %i –o %i –t Single –d 10 –id1 0.2 –id2 0.5

For user generate a certain copy of a sample, eg. Copy1.

python Cotu-generator.py -i BOP010001.fasta –o BOP010001 –t Multi –cp 1 -d 10 -id1 0.2 -id2 0.5

### In the beginning and ending region self-learned by Cotu-generator, 0.2 parameter will be used while in the middle region 0.5 parameter will be used for Cotu generation.

For users who want to generate multiple Cotu for all fasta files in a folder, we provide a cycle command: Please copy Cotu-generator.py into the folder before you run the command.

for %i in (*.fasta); do python Cotu-generator.py -i %i –o %i –t Multi –cp 1 –d 10 –id1 0.2 –id2 0.5

###Run all alignment fasta files for Cotu sequence generation.

The consensus sequences are output as a fasta file for each sample. Document name, sequence length and sequence depth were included in the name of consensus sequence.

**Cotu Master**

In order to facilitate researchers who are not good at bioinformatics, we packaged the sequence generation steps using Cotu method into “Cotu Master” (Supporting software.7z). Users can get final Cotu sequence very easily from a demltiplexed fastq file (with only one gene and one species in the fastq file and without artificial sequences).

**How to use Cotu Master**

Please download Cotu Master in https://github.com/YanleiLiu1989/Cotu-master and unzip usr.rar and tmp.rar in the Cotu Master folder or directly unzip Supporting software.7z.

Python script fun.py is written for combination of each core steps in Cotu generation. Open cmd.exe, change to Cotu Master working folder and input:

python Cotu-generator.py –help

You will see information below:

useage: python fun.py [options]

This is a script for generating Cotu directly from an original fastq file

-h, --help show this help message and exit

-cn, --copy_number COPY_NUMBER

This parameter is used for copy number of sequences

-s, --seq_number SEQ_NUMBER

This parameter is used for limiting the number of sequences in sequence alignment

--filelist FILELISE Fastq files list for Cotu generation

**Practical examples:**

We provide a test data in the Cotu Master package. Three test data in fastq formation were provided.

Run the Cotu-master to generate Cotu for the sample in the list.txt without the sequence number limitation in mafft alignment.

python fun.py --filelist list.txt

Run the Cotu-master to generate Cotu for the sample in the list.txt with the massive sequence number for mafft limited to 500.

python fun.py --filelist list.txt --seq_number 500

The run results of test data were also provided, researchers can compare the results after you operate using test data.

Besides, the package used many software already written by other researchers like Usearch and Vsearch. All parameter of each original steps (parameters in former step5-step7) can be changed in the fun.py file according to different researchers’ needs following the command explanation in each software manual.

For Vsearch command manual, please visit <https://github.com/torognes/vsearch>

For Usearch command manual, please visit <http://www.drive5.com/usearch/manual/>

For Cotu-generator.py manual, please visit <https://github.com/YanleiLiu1989/Cotu-master>

Multiple copy analysis is also achieved, please visit https://github.com/YanleiLiu1989/Cotu-master for more infromation.

**How to cite Cotu**

Liu Y, Xu C, Sun Y, Wu P, Chen X, Dong W, Yang X, and Zhou S. 2021. Method for quick DNA barcode reference library construction. Ecology and Evolution (submitted)
