# Supplementary material for: Method for quick DNA barcode reference library construction
Source: Ecol Evol. 2021 Aug 4;11(17):11627–38. doi: 10.1002/ece3.7788 (PMC8427591; doi:10.1002/ece3.7788)
Supplement: Supplementary file 13 — Supporting Document S2 [file ECE3-11-11627-s006.docx]

**Step by Step Instructions to the Cotu Method**

**for NGS Data Process**

Please read the whole text throughout before starting to work and install all programs necessary for analyses.

**Step 1: Quality control**

NGS QC Toolkit and an ActivePerl (https://www.activestate.com/products/perl/) would have been installed.

Windows+R🡪cmd🡪Enter🡪change to the working directory (in cmd.exe).

For paired end data using the command below:

perl IlluQC.pl -pe Illumina-read1.fastq Illumina-read2.fastq 2 A -statOutFmt 2 -p 4

For single end data using the command below:

perl IlluQC.pl -se Ion-Torrent-S5.fastq 1 A -statOutFmt 2 -p 4

After quality control, Illumina-read1-filtered.fastq, Illumina-read2-filtered.fastq and Ion-Torrent-S5-filtered.fastq were generated.

**Step 2: Merge of Illumina read 1 and read 2 (Skip this step for Ion Torrent S5 data)**

In this step, flash.exe is used to merge the paired end reads in Windows system using the command below:

flash.exe Illumina-read1-filtered.fastq Illumina-read2-filtered.fastq

The merged Illumina data is named Illumina-merged.fastq.

**Step 3: Data demultiplexing**

In this step, FASTX Toolkit is used in Linux. Install FASTX Toolkit first according to information at http://hannonlab.cshl.edu/fastx_toolkit/commandline.

We use fastx_barcode_splitter.pl for sample demultiplexing.

For example:

cat Illumina-merged.fastq | fastx_barcode_splitter.pl --bcfile mybarcodes.txt --bol --mismatches 2 --prefix /tmp/Illumina_ --suffix ".fastq"

FASTX Toolkit cannot recognize degenerate bases, so the degenerated bases have to be transferred into one type of bases. Mismatches parameters can be modified according to the numbers of degenerated bases.

**Step 4: Artificial sequence removal**

Artificial sequences are trimmed off using Geneious in Windows system or Cutadapt in Linux system.

In Geneious for example:

Data was imported into Geneious. The first 24 bases (10 bases of label and 14 bases of introducer) at both 5’ and 3’ ends are trimmed off using trim command. The priming sites are also trimmed according to the sequences of primers. The trimmed reads are exported in fastq format.

In Cutadapt for example:

Cut the first 24 bases at both 5’ and 3’ ends

Cutadapt --cut 24 --cut -24 -o Illumina-merged.trimmed.fastq Illumina-merged.fastq

Cut 5’ end artificial sequences

cutadapt -g [primer sequence] -O 5 -e 0 -o Illumina-merged.trimmed2.fastq Illumina-merged.fastq --minimum-length 35 --discard-untrimmed --info-file reads.adapter.txt

Cut 3’ end artificial sequences

cutadapt -a [primer sequence] -O 5 -e 0 -o Illumina-merged.trimmed2.fastq Illumina-merged.fastq --minimum-length 35 --discard-untrimmed --info-file reads.adapter.txt

**Step 5: Reads sorting** (Optional, only for genes of multiple copies, e.g., mixed samples or some nuclear genes)

In this step, vsearch.exe (https://github.com/torognes/vsearch) will be used. Reads are clustered using a similarity of 0.97~0.99 according to the variability of gene fragments. The directions of sequences are adjusted automatically and all files in a given directory are analyzed using the command below one by one. The python script split_msa.py can be downloaded at github (https://github.com/wpwupingwp/python/blob/master/split_msa.py). Take two copy gene and 0.97 similarity as example:

for %i in (*.fastq) do python split_msa.py %i 0.97

For samples with too many reads, in order to reduce the alignment burden, only a certain number of reads are selected for consensus creation. Users can define the selected number based on the whole and average amount of the sequencing data. The python script Seqpick.py can be downloaded in https://github.com/Mycroft-maker/ Seqpick.py.

for %i in (*.aln) do python Seqpick.py %i [number]

**Step 6: Alignment**

Mafft (https://mafft.cbrc.jp/alignment/software/) is used to align the reads. The following command is used to adjust the sequence directions, align all (sorted) files in a directory and export alignments as fas files.

If you choose Reads sorting, please use the command below:

for %i in (*deal.fasta);do mafft --reorder --adjustdirection --globalpair %i>%i.fas

If the Reads sorting was not chosen, please use the command below:

Convert fastq file into fasta file

for %i in (*.fastq) do vsearch.exe --fastq_filter %i –fastaout %i.fasta

Export alignments of fasta file

for %i in (*fastq.fasta);do mafft --reorder --adjustdirection --globalpair %i>%i.fas

**Step 7: Consensus sequence creation**

Amplicon sequencing by NGS platform often produces reads with missing ends. This type of uneven reads may lead to varying lengths of consensus sequences when using software like Vsearch. Besides, existing data analysis methods using representative sequence or imperfect consensus generating strategy which ignored the uneven reads at both end. Here we propose a Cotu method which makes more reasonable uses of reads at sites, especially in the regions of both ends, and creates consensus sequences based on the majority rule. The Cotu method takes both the sequencing depth and the numbers of bases at a site into consideration when creating a consensus sequence using a python script cotu-generator.py from the aligned reads. In order to guarantee the accuracy of consensus sequences, a minimum sequencing depth is given by users, for example, 10×. When processing two ends of an alignment, if more than 20% of a site have bases instead of gaps, the gaps are ignored and the majority rule is applied to bases at this site for creating a consensus base (the region range at two ends is self-leaned by Cotu-Generator). For the region in the middle of an alignment, both gaps and bases are considered and the simple majority rule (> 50%) is applied when creating consensus sequences (a gap or a base at a given site) depending on their numbers. The minimum sequencing depth, the percentage of bases to the missing data at both ends, and the simple majority rule can be changed by users according to the actual data. The command for this step is below:

for %i in (*.fas); do python Cotu-generator.py -i %i –o %i –t Single –d 10 –id1 0.2 –id2 0.5

The consensus sequences are output as a fasta file for each sample. Document name, sequence length and sequence depth were included in the name of consensus sequence.

-h, --help show this help message and exit

-i: Input file.

-o Output sample name

-t Type of copy [Single or Multi]

-cp The ranking of copy number

-d: The minimum sequencing depth, sample with sequencing depth lower than 5 will be dropt.

-id1: The first sequencing depth strategy, in a certain region of two ends, base number counted larger than the parameter given will ignore gaps and conducted the majority for consensus generation.

-id2: The second sequencing depth strategy, in a certain region of the middle, base number counted larger than the parameter given will ignore gaps and conducted the majority for consensus generation. If not, a gap will be produced in the certain position.

**Citation**

Liu Y, Xu C, Sun Y, Wu P, Chen X, Dong W, Yang X, and Zhou S. 2021. Method for quick DNA barcode reference library construction. Ecology and Evolution (submitted)
